# Interventions to improve obstetric emergency referral decision making, communication and feedback between health facilities in sub‐Saharan Africa: A systematic review

**DOI:** 10.1111/tmi.13747

**Published:** 2022-04-05

**Authors:** Cephas K. Avoka, Eve McArthur, Aduragbemi Banke‐Thomas

**Affiliations:** ^1^ Faculty of Public Health Ghana College of Physicians and Surgeons Accra Ghana; ^2^ Faculty of Epidemiology and Population Health London School of Hygiene and Tropical Medicine London UK; ^3^ Costello Medical London UK; ^4^ School of Human Sciences University of Greenwich London UK; ^5^ LSE Health London School of Economics and Political Science London UK

**Keywords:** emergency obstetric care, health systems, maternal health, referral, sub‐Saharan Africa

## Abstract

**Objective:**

The objective of the study was to review the evidence on interventions to improve obstetric emergency referral decision making, communication and feedback between health facilities in sub‐Saharan Africa (SSA).

**Methods:**

A systematic search of PubMed, Embase, Cochrane Register and CINAHL Plus was conducted to identify studies on obstetric emergency referral in SSA. Studies were included based on pre‐defined eligibility criteria. Details of reported referral interventions were extracted and categorised. The Joanna Biggs Institute Critical Appraisal checklists were used for quality assessment of included studies. A formal narrative synthesis approach was used to summarise findings guided by the WHO's referral system flow.

**Results:**

A total of 14 studies were included, with seven deemed high quality. Overall, 7 studies reported referral decision‐making interventions including training programmes for health facility and community health workers, use of a triage checklist and focused obstetric ultrasound, which resulted in improved knowledge and practice of recognising danger signs for referral. 9 studies reported on referral communication using mobile phones and referral letters/notes, resulting in increased communication between facilities despite telecommunication network failures. Referral decision making and communication interventions achieved a perceived reduction in maternal mortality. 2 studies focused on referral feedback, which improved collaboration between health facilities.

**Conclusion:**

There is limited evidence on how well referral interventions work in sub‐Saharan Africa, and limited consensus regarding the framework underpinning the expected change. This review has led to the proposition of a logic model that can serve as the base for future evaluations which robustly expose the (in)efficiency of referral interventions.

## INTRODUCTION

More than 295,000 maternal deaths and 1.9 million stillbirths resulting from pregnancy and childbirth complications occur globally every year, many of which are preventable [1, 2]. Sub‐Saharan Africa (SSA) alone accounts for two‐thirds of maternal deaths and 42% of stillbirths worldwide [1, 2]. Life‐saving interventions altogether referred to as emergency obstetric care (EmOC) are used in the management of complications of pregnancy and childbirth. These clinical and surgical interventions include the use of parenteral antibiotics, anticonvulsants and uterotonics, manual removal of placenta, removal of retained products, neonatal resuscitation and assisted vaginal birth. Two EmOC interventions, in particular, (caesarean section and blood transfusion) need to be managed at higher levels of care for which referrals are likely to play a significant role [3, 4].

**FIGURE 1:**
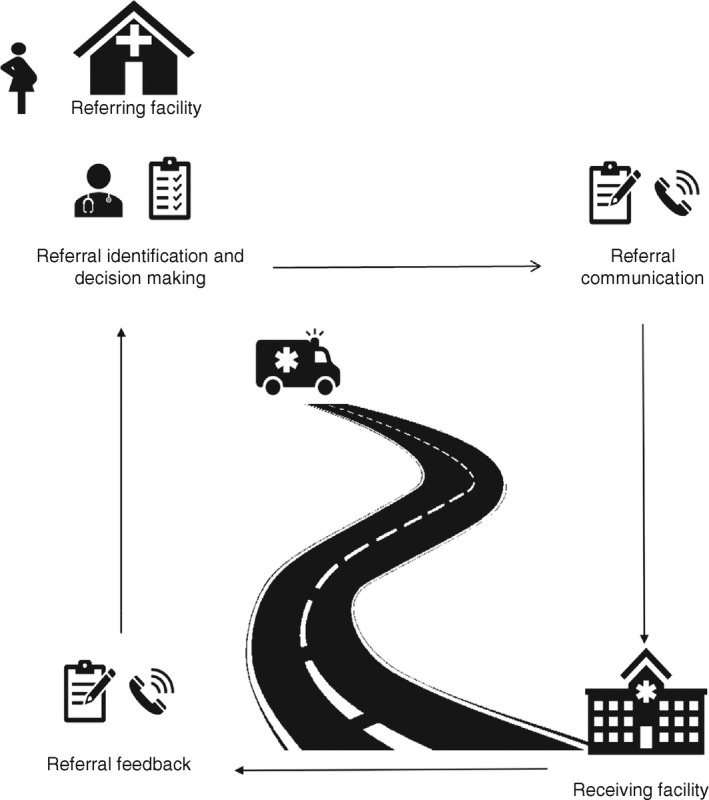
Referral identification/decision making, communication and feedback loop

According to the WHO, a referral is a ‘process in which a health worker at one level of the health system, having insufficient resources (medicines, equipment and skills) to manage a clinical condition, seeks the assistance of a better or differently resourced facility at the same or higher level to assist in or take over the management of the client's case’ [5]. A referral starts with the initiating facility (a health facility that decides to refer a client), where a client reports to and is assessed per the facility care protocol. When the decision to refer is made (referral decision making), the receiving facility (a health facility that agrees to receive and continue management of the client's condition) should be made aware (referral communication), so they can anticipate the client's arrival. After management, feedback should be provided to the initiating facility on the outcome of care and any follow‐up measures necessary (referral feedback). The provision of feedback completes the referral flow between these two facilities [5] (Figure 1).

Previous reviews in SSA have highlighted women's dissatisfaction with the referral process, poor coordination among staff, confusion over the clinical criteria for referral and poor referral documentation as barriers to accessing EmOC [6, 7, 8, 9, 10]. Although obstetric emergency referral is associated with decreased maternal mortality, poor liaison among health facilities in SSA results in unnecessary delays and affect EmOC service provision [6, 11]. Reviews that explored the effectiveness of obstetric referral interventions have mainly focused on strategies to reduce delays in reaching a referral facility [12, 13]. There is no review capturing interventions that minimise delays in referral when pregnant women arrive at health facilities. Delays by health workers in providing care or referring women after they arrive at a health facility increase the risk of maternal mortality [14]. This period aligns with the three key components of the referral process (decision making, communication and feedback) and has long been recognised as crucial gaps in the obstetric referral literature [15]. Our review describes and evaluates the available evidence on interventions to improve obstetric emergency referral decision making, communication and feedback between health facilities in SSA.

## METHODS

This systematic review was carried out following a PROSPERO registered protocol (Registration number: CRD42020222853).

### Search strategy

Using the Preferred Reporting Items for Systematic Reviews and Meta‐Analysis (PRISMA) approach [16], a systematic search of PubMed, Embase, Cochrane Central Register of Controlled Trials and CINAHL Plus was conducted. The search strategy used terms relating to "Emergency" OR "Referral" AND "Maternal" OR “Obstetric” OR "Childbirth Care", limiting to sub‐Saharan African countries and no limit on publication date. CA and AB‐T independently conducted the search, which was closed on 31 December 2020 to allow for analysis. (Table S1).

### Eligibility criteria

Using the Population‐Intervention‐Comparison‐Outcome (PICO) approach [17], articles were included if they were qualitative or quantitative interventional studies, assessing pregnant women requiring referral or healthcare personnel involved in referral, reporting on referral‐associated outcomes in SSA (Table S2). Studies that reported non‐obstetric emergency referrals, obstetric referral transport‐only interventions, commentaries, editorials and case reports were excluded from the review.

### Article screening, data extraction and synthesis

Titles and abstracts of all records identified were screened against the pre‐defined eligibility criteria by two reviewers (CA and AB‐T). Following this step, full texts of included articles were retrieved and screened against the eligibility criteria, by the same reviewers. Any disagreements between reviewers were resolved through discussion with the third reviewer (EMcA).

Data on study authors, year of publication, study design, participants involved, funding source, intervention and comparison groups, relevant intervention outputs, outcomes and indicators were extracted from all included studies into a pre‐designed grid in Microsoft Excel (Microsoft Corporation, Redmond, Washington, USA). Data were extracted by CA and verified by AB‐T. Evidence synthesis subsequently involved consolidating data extracted from included articles guided by the WHO referral systems flow [5]. Synthesis was conducted under the broad themes of identification/decision making, communication or feedback. Due to the heterogeneity of referral interventions and outcomes, the analysis and interpretation of the findings followed a formal narrative synthesis approach [18, 19].

### Quality assessment

Study quality was assessed by two reviewers (CA and AB‐T) using the Joanna Biggs Institute (JBI) critical appraisal checklists, with checklists selected based on study design [20, 21]. Disagreements were resolved through discussion with a third reviewer (EMcA). Each study was assessed using a scoring system developed based on the answer provided for each question on the checklist. A study scored 1 if the answer to the appropriate question was ‘yes’, 0.5 if the response was ‘partial’ and 0 if the response was ‘no’, with studies assessed as low (if they scored less than 50% overall), medium (50%–80%) or high quality (above 80%).

## RESULTS

### Included and excluded studies

**FIGURE 2:**
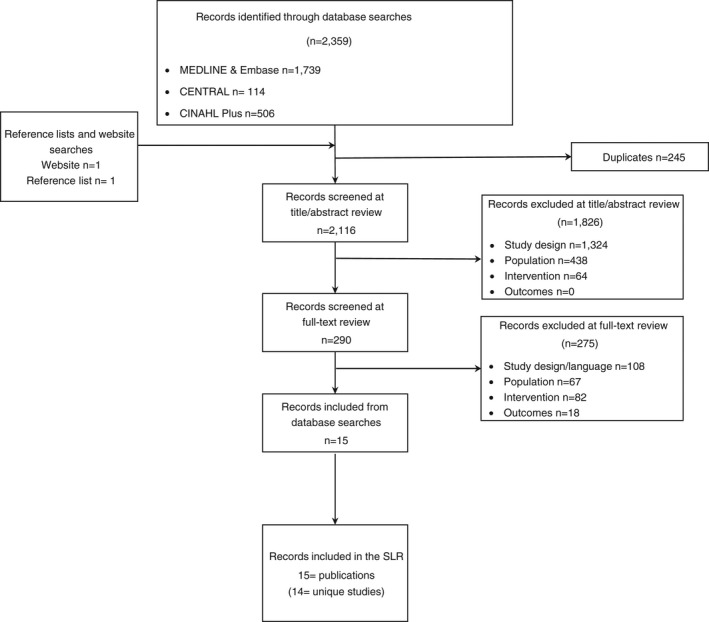
Preferred Reporting Items for Systematic Reviews and Meta‐Analysis (PRISMA) Chart

In all, 2359 records were obtained from database searches, with two records identified from website searches and reference lists. Across all sources, 14 unique studies met the eligibility criteria and were included for review (Figure 2).

### Characteristics of included studies

**FIGURE 3:**
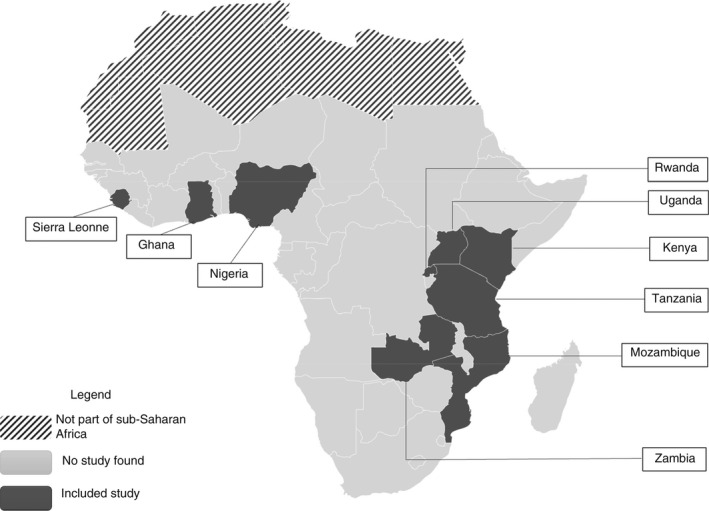
Map of Africa with included studies

Countries where included studies were conducted comprised Ghana [22, 23], Kenya [24], Mozambique [25], Nigeria [26], Rwanda [27, 28], Sierra Leone [29], Tanzania [30], Uganda [31, 32, 33, 34] and Zambia [35, 36] (Figure 3).

Of the 14 studies identified (Table 1), 8 were quantitative [25, 26, 27, 28, 29, 32, 33, 34, 35], including 2 RCTs [25, 32], and 6 were quasi‐experimental studies [22, 26, 28, 29, 33, 34, 35](Table 2). Five were qualitative [22, 23, 24, 30, 31, 36], with 1 mixed methods study [22] (Table 3).

**TABLE 1:** Summary of included studies

Characteristic	Number (Total = 14)	Percentage (%)
Study designs/methods		
Quantitative studies (including 2 randomised controlled trials)	8	57.1
Qualitative studies (including 1 mixed methods study)	6	42.9
Study interventions		
Studies reporting solely referral identification/decision‐making interventions	5	35.7
Studies reporting solely referral communication interventions	4	28.6
Studies reporting solely referral feedback interventions	0	0
Studies reporting referral identification/decision‐making and communication interventions	1	7.1
Studies reporting referral communication and feedback interventions	3	21.4
Studies reporting referral identification/decision‐making, communication and feedback interventions	1	7.1
Funding		
Studies funded by national governments	2	14.3
Studies funded by international institutions/organisations	10	71.4
Scope of intervention		
Multinational level	1	7.1
National level	2	14.3
Regional/provincial level	5	35.7
District level	5	35.7
Rural level	1	7.1
Quality assessment		
High quality	7	50
Medium quality	6	42.9
Low quality	1	7.1

**TABLE 2:** Summary of the quantitative studies included in this review

Author (year), country	Study design (method)	Participants	Intervention	Funding	Comparison	Relevant outcomes		Strength of evidence
						Intervention group	Comparison group	
Akpala (1994), Nigeria [26]	Quantitative (Uncontrolled before‐after study)	Trained TBAs Untrained TBAs	43 TBAs trained on identification of high‐risk pregnancies and deliveries for referral to health institutions	Sokoto state government	31 Untrained TBAs	Calculated average percentage detection of high‐risk pregnancies among trained TBAs = 78%	Calculated average percentage detection of high‐risk pregnancies among untrained TBAs = 22%	*p* = 0.001
Henry (2018), Zambia [35]	Quantitative (Controlled before‐after study)	Non‐clinical community‐based volunteers referred to as Safe Motherhood Action Groups (SMAGs)	Cohort of women who delivered during the Saving Mothers Giving Life (SMGL) intervention to improve emergency referral response via functional radio systems in Kalomo.	Bill and Melinda Gates Foundation Ministry of Health	Cohort of women who delivered before SMGL intervention and who live in adjacent and socio‐demographically similar districts	Absolute percentage difference (before and after intervention) in facility‐based birth = +9.8% (95% CI 7.4%, 12.2%)	Absolute percentage difference (before and after intervention) in facility‐based birth = +0.2% (95% CI −1.4%, 1.7%)	*p* < 0.001 (reported for intervention group)
						Absolute percentage difference (before and after intervention) in attendance by skilled‐based provider = 5.5% (95% CI 3.0%, 8.0%)	Absolute percentage difference (before and after intervention) in attendance by skilled‐based provider = −0.4% (95% CI −2.0%, 1.2%)	*p* < 0.001
Kanyesigye (2019), Uganda [32]	Quantitative (RCT)	Health facility workers	Health centres randomised to receive a mobile phones and recharge credit to make pre‐referral phone calls to a dedicated number at a major referral hospital. Prior to randomisation, health centres had similar characteristics. Home visits by Village Health Teams (VHTs) in intervention communities	Not indicated	Health centres in the control group who did not receive the mobile phone and recharge credit	Percentage of pre‐referral phone calls made = 66.67%	Percentage of pre‐referral phone calls made = 5.56%	*p* = 0.001
						Percentage of women who had a caesarean section as outcome of pregnancy = 60.98%	Percentage of women who had a caesarean section as outcome of pregnancy = 39.02%	*p* = 0.022
Leigh (1986), Sierra Leone [29]	Quantitative (Non‐randomised trial)	Maternal and Child Health Aides (MCHAs)	30 MCHAs identified by random selection and trained to use of partograph to identify high‐risk pregnancy or cases for referral.	Not indicated	30 midwives randomly selected to undergo similar training	Percentage of women referred when cervical dilatation curve crosses action line = 83%	Percentage of women referred when cervical dilatation curve crosses action line = 90%	*p* > 0.2
						Proportion of women who had caesarean section at birth = 3/15	Proportion of women who had caesarean section at birth = 11/20	*p* < 0.05
Mucunguzi (2014), Uganda [34]	Quantitative (Controlled before‐after study)	Health facility workers	Health facilities in intervention districts provided with a mobile phone and airtime to communicate with ambulance team and referral facility.	Italian NGO (CUAMM)	Neighbouring district which is similar to intervention district in demographics, culture, history and economic activities and which did not receive the intervention	Absolute change in mean annual caesarean section rate = 0.87–1.66	Absolute change in mean annual caesarean section rate = 0.50–0.56	*p* = 0.034
						Absolute number of hospital births = 1090–1646	Absolute number of hospital births = 1776–1810	Not reported
Ruton (2018) [28] and UNICEF (2016) [27], Rwanda	Quantitative (Interrupted time series analysis)	Community Health Workers (CHWs)	Use of mobile phones to quickly link mothers to emergency obstetric care through alerts that notify ambulance services (Rapid‐SMS) Interrupted time series analysis using database of text messages sent by CHWs 24 months after start of intervention	UNICEF	Interrupted time series analysis using database of text messages sent by CHWs for 14 months before start of intervention	Percentage relative increase in number of facility deliveries over 1 year = 17.6%	No change (data not provided)	*p* < 0.001
Santos (2020), Uganda [33]	Quantitative (Uncontrolled before‐after study)	Midwives	Use of triage checklist to prompt providers to perform clinical assessment and guide referrals (Phase 2) Use of focussed ultrasound scan to assess foetus and refer if abnormal (Phase 3) Provision of mobile airtime to support communication between primary health centre and district hospital. Phased intervention. Phase 2 (triage checklist) and Phase 3 (triage checklist + focussed ultrasound) compared with Phase 1 (standard of care)	Bill and Melinda Gates Foundation	Standard of care guidance to refer to higher care any conditions of interest unless birth is imminent (Phase 1)	Referral rates between primary health centre and district hospital: Phase 2 (4.3%) and Phase 3 (35.5%)	Referral rates between primary health centre and district hospital: Phase 1 (2.2%)	*p* < 0.001
						Rate of intent to refer high‐risk pregnancies among midwives: Phase 3 (41.3%)	Rate of intent to refer high‐risk pregnancies among midwives: Phase 1 (14.4%)	*p* < 0.001
						Incidence of maternal complications: Phase 2 (5.6%) and Phase 3 (4.4%)	Incidence of maternal complications: Phase 1 (1.65%)	*p* = 0.407 (Phase 2 vs. Phase 1) *p* = 0.116 (Phase 3 vs. Phase 1)
						Incidence of foetal complications: Phase 2 (3.2%) and Phase 3 (3.7%)	Incidence of foetal complications: Phase 1 (2.9%)	*p* = 0.765 (Phase 2 vs. Phase 1) *p* = 0.465 (Phase 3 vs. Phase 1)
						Diagnostic sensitivity of the checklist for any maternal condition: Phase 2 (65.3%) and Phase 3 (73.7%) Diagnostic sensitivity of the checklist for any foetal condition: Phase 2 (57.1%) and Phase 3 (62.5%)	Diagnostic sensitivity of the checklist for any maternal condition: Phase 1 (57.6%) Diagnostic sensitivity of the checklist for any foetal condition: Phase 1 (10.0%)	*p* = 0.401 (Phase 2 vs. Phase 1) *p* = 0.204 (Phase 3 vs. Phase 1) *p* < 0.001 (Phase 2 vs. Phase 1) *p* < 0.001 (Phase 3 vs. Phase 1)
Sevene (2020), Mozambique [25]	Quantitative (RCT)	Community Health Workers	Use of mobile health application with pictogram as visual prompts to observe women and rule out emergency conditions that would warrant immediate referral to facility. Health clusters randomised into intervention and control groups.	Bill and Melinda Gates Foundation	Women in control groups received routine antenatal care provided by nurses and doctors.	Maternal deaths per 1000 identified pregnancies = 0.2%	Maternal deaths per 1000 identified pregnancies = 0.1%	*p* = 0.26
						Maternal morbidity per 1000 identified pregnancies = 9.2%	Maternal morbidity per 1000 identified pregnancies = 9.6%	*p* = 0.48
						Percentage of stillbirths = 2.5%	Percentage of stillbirths = 2.3%	*p* = 0.04
						Percentage of early neonatal deaths = 2.3%	Percentage of early neonatal deaths = 2.1%	*p* = 0.56
						Percentage of facility births=67.3%	Percentage of facility births = 74.2%	*p* = 0.71

Abbreviations: CHW, community health worker; RCT, randomised control trial; TBA, traditional birth attendant.

**TABLE 3:** Summary of the qualitative studies included in this systematic review

Author (year), country	Participants	Funding	Methods	Intervention	Relevant outcomes
Amoakoh‐Coleman (2019), Ghana]	Health facility workers (midwives) Health facility managers	World Health Organization TDR Postdoctoral grant	FGDs IDIs Non‐participant observations	Training on inter‐facility communication Sharing client information using referral notes Provision of mobile phones and call credit Monthly phone reminders and onsite visits to discuss and emphasise feedback on referrals Designating task of inter‐facility communication to team of representatives from facilities	Absolute increase in the number of referrals between intervention and comparison groups by 41% Absolute increase in the number completed referral notes by 17% Use of referral forms to promote inter‐facility communication of referrals
Banke‐Thomas (2019), Kenya (Banke‐Thom as et al., 2020)	Health facility workers Trainers and training organisers Officials from Ministry of Health	United Kingdom Department for International Development (DFID)	KIIs FGDs PIs	Hands‐on ‘skills and drills’ training for Health facility professionals to improve knowledge and skills in identifying high‐risk obstetric cases	Improved communication with clients leading to increased trust and utilisation of facility services Perceived reduction in morbidity and mortality among pregnant mothers and their babies
Dillip (2017), Tanzania [30]	Accredited Drug Dispensing Outlets (ADDOs) CHWs Health facility workers	Bill and Melinda Gates Foundation	IDIs FGDs	ADDOs and CHWs trained to identify high‐risk cases and refer clients to health facilities Training encouraged communication between ADDOs, CHWs and Health facility workers HFWs communicate with ADDOs to dispense drugs not available. Health facility workers provide feedback to CHWs on cases managed	Increased level of satisfaction among women on referral due to quicker access to treatment and priority given at health facility Increased collaboration between ADDOs, CHWs and Health facility workers More clients report to facility due to increased detection of danger signs Perceived reduction in deaths of women and children
Jacobs (2018), Zambia [36]	Safe Motherhood Action Groups (SMAGs) Health facility workers	Africa Union	FGDs IDIs	Standard training programme to empower SMAGs with skills to identify high‐risk cases in community and refer to health facility	Increase in access to skilled attendance at birth
Mangwi Ayiasi (2015), Uganda [31]	Village Health Teams (VHTs) Health facility workers	Institute of tropical medicine, Antwerp	FGDs KII	VHTs make home visits and communicate potential referrals with professional health workers using mobile phones provided. Professional health workers provide feedback to VHTs through phone calls	Immediate feedback on referral consultation sometimes prevents travel to facility which reduces cost associated with the journey
Amoakoh (2019), Ghana [23]	Health facility workers	Netherlands foundation for scientific research	FGDs KIIs	Use of emergency protocols to guide management and referral of high‐risk obstetric cases Provision of personal and facility‐based mobile phones with top‐up credit to promote communication between facilities at different levels in an administrative district	Acceptance of intervention and satisfaction with its use

Abbreviations: CHW, community health worker; EmOC, emergency obstetric care; FGD: focussed group discussion; HCP, health facility professional; HFW, health facility worker; IDI, in‐depth interviews; KII, key informant interviews; PI, paired interviews.

Five studies reported solely on interventions to improve referral identification and decision making [24, 25, 26, 29, 36], and another 4 focused solely on referral communication interventions [27, 32, 34, 35]. No study focused exclusively on referral feedback interventions. Five studies had multi‐component interventions, 1 on referral identification and communication [33], another 3 on both referral communication and feedback [22, 23, 31], and 1 captured all three components [30] (Table 1).

Two studies [29, 32] were sponsored by national governments, 2 did not report funding and 10 other studies received funding from international and multilateral organisations such as the Bill and Melinda Gates Foundation [30, 33, 35]. Two studies focused on interventions implemented at the national level [24, 28], one intervention was multinational [35], with the remaining interventions focused on rural [26], district [22, 31, 33, 34, 36] and regional or provincial levels [23, 25, 29, 30, 33] (Table 1).

### Quality assessment of included studies

Of the 14 included studies, 7 were assessed to be of high quality (5 quantitative studies [25, 28, 33, 34, 35] and 2 qualitative studies [24, 30]; 6 studies were assessed as medium quality) (2 quantitative [26, 32] and 4 qualitative [22, 23, 31, 36], and 1 quantitative study was assessed to be of low quality [31]). Most of the quantitative studies (which were predominantly before‐after studies) scored lowest on providing multiple measurements of outcomes before and/or after the intervention, whilst all qualitative studies failed to provide a statement locating the researcher culturally or theoretically (Table 4).

**TABLE 4:** Quality assessment of included studies

Quasi‐experimental studies							Randomised controlled trials			Qualitative studies						
JBI checklist criteria	Henry, 2018	Leigh, 1986	Akpala, 1994	Mucunguzi, 2014	Ruton, 2018	Santos, 2020	JBI checklist criteria	Kanyesigye, 2019	Sevene, 2020	JBI checklist criteria	Amoakoh‐Coleman, 2019	Banke‐Thomas, 2019	Dillip, 2017	Jacobs, 2018	Mangwi Ayiasi, 2015	Amoakoh, 2019
Is it clear in the study what is the ‘cause’ and what is the ‘effect’ (i.e. there is no confusion about which variable comes first)?	1	1	1	1	1	1	Was true randomisation used for assignment of participants to treatment groups?	1	1	Is there congruity between the stated philosophical perspective and the research methodology?	1	1	1	0.5	0.5	0
Were the participants included in any comparisons similar?	1	0	0.5	1	1	1	Was allocation to groups concealed?	N/A	N/A	Is there congruity between the research methodology and the research question or objectives?	1	1	1	1	1	1
Were the participants included in any comparisons receiving similar treatment/care, other than the exposure or intervention of interest?	1	0.5	0.5	0.5	1	1	Were treatment groups similar at the baseline?	1	1	Is there congruity between the research methodology and the methods used to collect data?	1	1	1	1	1	1
Was there a control group?	1	1	1	1	0	0	Were participants blind to treatment assignment?	N/A	N/A	Is there congruity between the research methodology and the representation and analysis of data?	1	1	1	1	0	1
Were there multiple measurements of the outcome both pre and post the intervention/exposure?	0	0	0	1	1	0	Were those delivering treatment blind to treatment assignment?	N/A	N/A	Is there congruity between the research methodology and the interpretation of results?	0.5	1	1	1	1	1
Was follow‐up complete and if not, were differences between groups in terms of their follow‐up adequately described and analysed?	0.5	0.5	0.5	1	1	1	Were outcomes assessors blind to treatment assignment?	N/A	N/A	Is there a statement locating the researcher culturally or theoretically?	0	0	0	0	0	0
Were the outcomes of participants included in any comparisons measured in the same way?	1	1	0.5	1	1	1	Were treatment groups treated identically other than the intervention of interest?	1	1	Is the influence of the researcher on the research, and vice‐ versa, addressed?	0	1	0.5	0	0	0
Were outcomes measured in a reliable way?	0.5	0	0.5	0	0.5	1	Was follow‐up complete and if not, were differences between groups in terms of their follow‐up adequately described and analysed?	1	1	Are participants, and their voices, adequately represented?	1	1	1	1	1	1
Was appropriate statistical analysis used?	1	0	0.5	1	1	1	Were participants analysed in the groups to which they were randomised?	0.5	1	Is the research ethical according to current criteria or, for recent studies, and is there evidence of ethical approval by an appropriate body?	1	1	1	1	1	1
							Were outcomes measured in the same way for treatment groups?	1	1	Do the conclusions drawn in the research report flow from the analysis, or interpretation, of the data?	1	1	1	1	1	1
							Were outcomes measured in a reliable way?	1	1							
							Was appropriate statistical analysis used?	0	1							
							Was the trial design appropriate for the topic, and any deviations from the standard RCT design accounted for in the conduct and analysis?	0	0.5							
**Total score (of maximum 9)**	**7**	**4**	**5**	**7.5**	**7.5**	**7**	**Total score (of maximum 9)**	**6.5**	**8.5**	**Total score (of maximum 10)**	**7.5**	**9**	**8.5**	**7.5**	**6.5**	**7**
**Total score (in percentages)**	**78**	**44**	**56**	**83**	**83**	**78**		**72**	**94**		**75**	**90**	**85**	**75**	**65**	**70**

Note

Grading: Yes (1), Unclear (0.5), No (0); Quality assessment: High (>80%‐100% (Green)), Medium (50%‐80% (Yellow)), Low (<50% (Red)).

### Referral interventions and outcomes

#### Referral identification and decision making

Of all seven studies that reported on interventions to improve referral identification and decision making, training programmes of durations ranging from 3 days to 5 days were popular [23, 24, 25, 26, 29, 30, 33, 36]. A 3‐day seminar on identifying high‐risk pregnancy through the use of partographs (with the alert line drawn in the active phase of labour) by maternal and child health aides (MCHAs) in Sierra Leone resulted in similar referral rates between MCHAs and trained midwives [29] (Table 2), whilst a 5‐day ‘skills and drills’ training combined simulation‐based medical education with deliberate practice among health facility workers in Nairobi, Kenya [24] (Table 3). Another study explored the outcomes of a training programme targeted at TBAs in Nigeria, by comparing their knowledge and referral practices with those of untrained TBAs, with results showing that trained TBAs on average had rates of detecting high‐risk pregnancies (such as labour cases lasting more than 24 h) as high as 56.0% more than untrained TBAs [26]. In Zambia, community health workers known as Safe Motherhood Action Groups were trained to identify danger signs and encourage women to receive skilled birth attendance [36] (Table 2). Training interventions in general were deemed to have led to an increase in knowledge and skills of health workers to effectively identify obstetric emergencies which warrant referral to a higher‐level facility [24, 26, 29, 33], increased rates of deliveries in a health facility [24, 25] and perceived reduction in maternal morbidity and mortality [24, 25, 30, 33]. However, these training interventions did not always result in anticipated outcomes, as is seen in Nigeria, where following a TBA training programme, trained and untrained TBAs reported similar referral rates for conditions such as post‐partum haemorrhage (PPH) [26]. Additionally, trained male CHWs had fewer referrals because women in the community preferred to be examined and subsequently referred by female CHWs instead [36].

Other interventions involved the use of a triage checklist with focused obstetric ultrasound by midwives in Uganda, which resulted in an increase in referrals by 31.2% compared with the standard of care [33], and the use of pictograms on mobile phones as visual prompts for pre‐eclampsia risk classification by CHWs in Mozambique, which allowed them to identify emergency referrals based on blood pressures measurements [25] (Table 2). Although the obstetric ultrasound played a catalytic role in increasing the sensitivity of the triage checklist for detecting high‐risk pregnancies, this resulted in higher rates of referrals due to more false positives, and an increased burden on higher‐level facilities as reported by health facility workers [33]. Also, the use of pictograms on mobile phones did not result in a significant reduction in maternal and neonatal deaths which the authors attributed to the small number of clusters in the study and deficiencies in facility‐based care [25]. Another study in Tanzania reported that having danger signs posters on the walls of health facilities aided in the detection of high‐risk cases and the explanation of these risks to pregnant women, which encouraged them to adhere to the referral process [30].

#### Referral communication

The use of mobile phones was the most common intervention to improve referral communication. Mobile phones were used to communicate referrals between healthcare personnel [31] and between health facilities. In Ghana, separate mobile phones were provided for healthcare workers and health facilities [23] whilst in Zambia, a functional radio system was provided to the health facility [35]. In Uganda, mobile phones were provided to community health workers called village health teams (VHTs) who visited women in their homes and communicated any potential referrals with health facilities [31] (Table 3). Mobile phones were mostly used by health workers and facilities to share information about the client being referred (with referral facilities) [22], to make enquiries about actions to be taken before a client is referred, [23] and to notify ambulance services of an impending referral through short message service alerts [27]. Several outcomes were reported from these interventions including an increase in absolute percentage of deliveries in health facilities, an increase in communication between facilities concerning referrals in general, an increase in rates of emergency caesarean section as an indicator for access to emergency care, and an increase in ambulance response rates [28, 34, 35]. However, some challenges with the use of mobile phone interventions included calls being ‘*received with anger from referral facilities’*, a failure of telecommunication networks and a malfunction of phones with time [23, 31]. In some facilities, health workers reported being unaware of the existence of the mobile phone used for referrals whilst the relatively older midwives reported being unfamiliar with texting as a mode of communication [23] (Table 3).

Referral forms were used as an intervention to promote referral communication in studies conducted in Ghana and Tanzania [23, 30] (Table 3). CHWs were reported to place a high value on the use of these forms [30], and there was no discrimination in the use of these forms for emergency and non‐emergency referrals [22]. The use of referral forms was associated with an increase in overall rate of referrals by about 41% [22], increased collaboration between health facilities, increased satisfaction with the referral process among health workers and pregnant women and a perceived reduction in maternal death [23, 30]. Some challenges reported with the use of referral forms included a stock outs of forms, forms being incomplete with insufficient details about the client's condition and initial management [22, 30]. In a study conducted in Ghana, some referred clients went home and later reported without the form, or refused to show the referral forms because they wanted a different opinion at the referral facility whereas in Uganda, personnel at referral facilities were reported to be absent when women arrived with these referral forms [22, 31]. Training was a key component of all the included referral communication interventions.

#### Referral feedback

Three studies reported on outcomes of referral feedback interventions [22, 30, 31], and these involved health facility workers providing feedback on referrals to CHWs, VHTs and Accredited Drug Dispensing Outlets through the use of mobile phones and referral forms [22, 30, 31]. All these interventions were complimented by training programmes which resulted in improved collaboration between health facilities and health workers involved in the referral process as the main outcome reported [22, 30]. Referral forms were mostly used to provide feedback if the initial referral was communicated using a referral form, as clients were directed to bring a piece of the referral form back to the CHW after visiting the receiving facility [30].

## DISCUSSION

In this review, we set out to describe and evaluate the available evidence on interventions aimed at improving the obstetric emergency referral process in SSA through its component steps. Our findings show that only nine of 46 countries in SSA (19.5%) have at least one study exploring issues of improvement in referral processes, with Uganda being the most widely studied (Four studies). Across the sub‐region, only two studies have been RCTs. Most interventions have focused on referral communication (50%) whilst feedback has been given the least focus (21%). Most of the interventions (86%) were implemented with support from foreign donors, with many implemented at the local level and on a small scale. Training and triage checklist were widely used for referral identification and decision making and shown to be effective in improving referral process indicators and some perceived reduction in referral outcomes. Mobile phones are most widely used for communication with all evaluation limited to process indicators. For feedback, limited evaluation was conducted but some qualitative evidence of effectiveness for process improvements have been noted.

In terms of study design and quality of published studies, only two were RCTs, which are generally seen as the gold standard for impact evaluation of interventions [37]. Whilst other study designs offer very useful information regarding the impact of the interventions, more RCTs need to be encouraged to ensure that the evidence base for potential scale‐up of interventions can be robust. The main reason for low quality for quantitative studies was for providing multiple measurements of outcomes before and after the intervention. Consensus is needed around this underpinned by a theory of change that clearly maps how these various interventions clearly link to the desired effect on outcomes [38]. For qualitative studies, the low point for quality assessment was with non‐inclusion of a statement describing positionality of the researcher. This is very important in qualitative research but more so for intervention‐based qualitative studies in which those who are implementing are also typically the ones evaluating [39].

For specific interventions being implemented, training of varying length of days were generally reported as being effective for improving identification and decision making. Training such as these have been shown to be cost‐effective [40] and guarantee value for money across multiple stakeholders [41]. However, as our review shows these trainings need to be targeted at the right personnel who have been motivated and equipped sufficiently to be able to make the right decision on referral for the women. For example, although training of TBAs resulted in higher rates of detection, it did not improve referral rates for PPH [28]. Evidence suggests that there are other factors that contributed to lack of improvement in referral rates including reported abuse by skilled health personnel and perception that obstetric fistula is a disease caused in the hospital [42]. In addition, trainings to inform decision making for referral have been reported to lead to higher rates of referrals being perceived as an increased burden on referral facilities. Whilst there may be multiple reasons for this feeling of burden, it brings to question the purpose of the training. Is it to increase referral or to optimise referral? Increased referral should not be part of outcomes of judging effectiveness of referral interventions without some judgement of the appropriateness of the referral itself. Other interventions such as triage checklist combined with ultrasound in Uganda and danger sign posters in Tanzania have been used for detection of high‐risk cases for foetuses and mothers respectively. However, the evidence on how well these have worked is limited at the present time.

Regarding communication, mobile phones have been commonly used, with varying modalities in how they were deployed including giving them directly to health workers or placing them centrally in the health facility and used to communicate with other health workers, ambulance services or health facilities. Implementation Science research is needed to properly understand which implementation approach/design works better in these settings. Outcomes reported were a mix of process and outcome indicators including increased communication, increased ambulance response rate and increased caesarean sections. However, mobile technology may not be the magic bullet in addressing the communication between health workers and facilities, as there are many challenges that limit its effectiveness. Challenges with the use of mobile phones include location of the intervention (network challenges), sustainability of such interventions (maintenance of phones and phone credit usage) and safety of client data with personal use. A recent review highlighted the need to apply information technology to address barriers in the referral system [10]. The other commonly used method to address communication was referral forms. However, stock outs and incomplete filling of forms hampered communication. As a communication tool, there is a need to balance the merits and demerits of referral notes vs. mobile technology. Mobile phone/technology ensures the referral facility is ready and anticipating arrival of clients. It also averts the risk of depending on clients to serve as the medium through which referral communication is completed (barriers posed by women hiding or misplacing forms). However, it is cheaper to use referral forms. The focus must be on cost‐effectiveness of either intervention, considering the feasibility of its intervention and ease of use among health facility workers.

For referral feedback, there is a paucity of evidence to make any credible assessments here. However, it is worth highlighting that discussing and evaluating the referral process is as important as evaluating the management of the client. Referral feedback completes the continuum of quality care for which the referral was initiated and is a key component of a functional referral system [43]. Additionally, feedback of referral ensures a continuous loop of communication between health facility workers and health facilities, resulting in an improved collaboration within the health system as was highlighted in our review.

**FIGURE 4:**
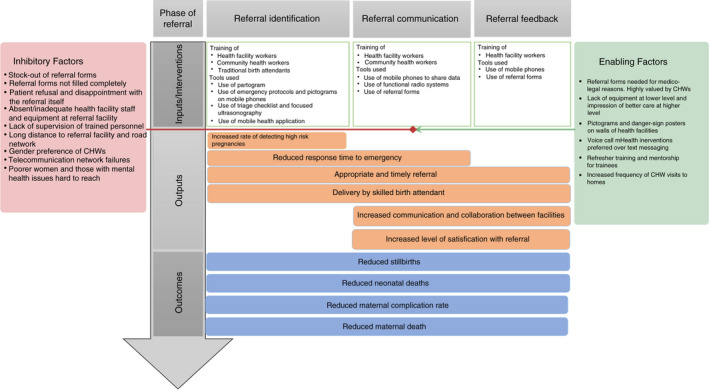
Logic model for referral interventions

Building on best practice, knowledge garnered from this review and the broad literature on referral [15, 44], we propose a logic model that can inform the future evaluation of referral interventions (Figure 4). Logic models built around a theory of change have been used to understand complex programmes and improve health outcomes and have also been proposed to be valuable in the systematic review process [38]. Theory‐of‐change methods have been employed in understanding referral systems as well as proposing frameworks for reducing delays in accessing emergency obstetric care [45, 46]. Logic models outline how a programme is designed to achieve its intended outcomes, including the evidence underpinning the complex pathways of interventions [47]. In this proposed logic model, we combine the interventions to improve referral processes, process indicators and outcomes contained within this review with a contextual understanding of how referral processes work in sub‐Saharan Africa to propose a theory‐based approach to studying, understanding and improving referral process for obstetric emergencies. Quality indicators that reflect a properly done referral, as recommended by experts include process indicators such as ‘appropriate referral’, and outcome indicators as ‘reduced maternal complication’ or ‘averted maternal death’, including the number of beneficiaries of such interventions [48, 49].

### Strengths and limitations

Strengths of the review include use of a pre‐specified systematic review protocol, the searching of multiple electronic databases, supplemented by hand‐searching of grey literature. As far as we know, this is the first review of its kind to assess these understudied components of the obstetric emergency referral process in sub‐Saharan Africa. Judging from how recent the studies are, the outcomes of these component interventions could contribute to broader assessments of referrals in reducing delays to accessing emergency obstetric care. This review also highlights key barriers and enablers of obstetric emergency referral interventions, providing contextually relevant recommendations for future research and practice. However, a key limitation to consider is that we have only included referral interventions that were published in peer‐reviewed literature. It is likely that there are some unpublished referral interventions that exist in national and sub‐national reports that are not available in the public domain and thus might have been excluded. Additionally, most interventions reported positive outcomes which could be an indication of reporting as well as publication bias [50]. We attempted to mitigate this by reviewing websites of organisations working at country level to improve maternal health systems and we believe that our assessment point more to the relatively small numbers of such studies rather than our inability to access them.

## CONCLUSION

This review has shown the paucity of literature focused on interventions to improve the referral process in SSA. Whilst a broad spectrum of interventions has been implemented across the processes of decision making, communication and feedback, there is limited evidence on how well these interventions have worked, especially how intervention outputs relate to the goal of reducing poor pregnancy outcomes for mothers and babies. For interventions that were deemed effective, what are the contextual factors that influenced these? If progress is going to be made, bridging this know‐do gap will be critical. Hopefully, the logic model proposed in this review along with suggested indicators to map the various steps will serve as a base for future evaluations.

## Supporting information

Appendix S1
