# Supplementary material for: Interventions to improve obstetric emergency referral decision making, communication and feedback between health facilities in sub‐Saharan Africa: A systematic review
Source: Trop Med Int Health. 2022 Apr 5;27(5):494–509. doi: 10.1111/tmi.13747 (PMC9321161; doi:10.1111/tmi.13747)
Supplement: Supplementary file 1 — Appendix S1 [file TMI-27-494-s001.docx]

**Supplementary Table S1: Full search strategy**

The search strategy was based on terms related to Emergency OR Referral, Maternal OR Childbirth Care, AND sub-Saharan Africa as shown in Table 1. MEDLINE, EMBASE, Cochrane Central Register of Controlled Trials (CENTRAL), and Cumulative Index of Nursing Allied Health Literature (CINAHL) Plus were searched for articles published in English language with no date limitation. Duplicates were identified and removed. The Cochrane Database of Systematic Reviews (CDSR) was also searched only at title/abstract stage for relevant systematic reviews. Subsequently, a reference list checking of these systematic literature reviews on obstetric referrals was conducted to identify any additional relevant articles that might have been missed. Conference abstracts of the International Federation of Obstetrics and Gynaecology from 2018 to date were also searched, as well as websites of the United Nations Population Fund (UNFPA), United Nations International Children’s Emergency Fund (UNICEF), United States Agency for International Development (USAID), the World Bank, and the Safe Motherhood Initiative. Following title and abstract screening, full texts of included articles were assessed to identify those that investigated relevant referral interventions and associated outcomes.

**Table 1: Search terms used to search MEDLINE and Embase on the OVID platform simultaneously, and then adapted for other databases.**

| Term Group | # | Searches |
| --- | --- | --- |
| Emergency referral terms |  | exp "referral and consultation"/ |
|  |  | Patient transfer/ |
|  |  | (emergenc* adj5 (refer* or communicat* or healthcare or health-care or consult* or intervention*)).ti,ab,kw. |
|  |  | ((doctor* or nurse* or obstetr* or midwife* or midwive* or centre* or facilit* or attendant* or "health centre" or "health facility") adj3 (refer* or emergenc* or communicat* or feedback)).ti,ab,kw. |
|  |  | ((phone or radio or mhealth or "mobile health" or mobile or text or SMS or telemedicine) adj5 (healthcare or "health care" or hospital* or clinic* or "health centre" or healthwork* or refer* or transport* or emergenc* or alert*)).ti,ab,kw. |
|  |  | or/1-5 |
| Maternal/ childbirth care terms |  | maternal health services/ or maternal-child health services/ or maternal death/ or maternal mortality/ or labor complications/ |
|  |  | delivery, obstetric/ or extraction, obstetrical/ or labor, obstetric/ |
|  |  | (obstetric or labour or labor or childbirth or "child birth").ti,ab,kw. |
|  |  | (pregnan* adj (wom?n or mother or care*)).ti,ab,kw. |
|  |  | or/7-10 |
|  |  | exp "Africa South of the Sahara"/ |
| Geographic limit |  | (Angola or benin or botswana or "Burkina fasso" or burkina faso or Burundi or Cameron or cameroon or "Cape Verde" or cabo verde or "Central African Republic" or Chad or Comoros or Congo or "democratic republic of the congo" or zaire or "ivory coast" or "cote d’ivoire" or Djibouti or "Equatorial Guinea" or Eritrea or Ethiopia or Gabon or Gambia or Ghana or Guinea or Guinea-Bissau or "guinea bissau" or Kenya or Lesotho or Liberia or Madagascar or Malawi or Mali or Mauritania or Mauritius or Mozambique or Namibia or Niger or Nigeria or Rwanda or "Sao Tome" or "sao tome and principe" or Senegal or Seychelles or "Sierra Leone" or Somalia or "South Africa" or "South Sudan" or Sudan or Swaziland or eswatini or Tanzania or Togo or Uganda or Zambia or Zimbabwe or "western africa" or africa, western or "eastern africa" or africa, eastern or "southern africa" or africa, southern or "central africa" or africa, central or "africa south of the sahara" or "sub-saharan africa" or "subsaharan africa").ti,ab,sh,kf. |
|  |  | or/12-13 |
| Exclusion terms |  | ("conference abstract" or "conference review").pt. |
|  |  | limit 15 to yr="1974-2018" |
|  |  | exp animals/ not exp humans/ |
|  |  | (comment or editorial or "case reports").pt. |
|  |  | (case stud$ or case report$).ti. |
|  |  | historical article/ |
|  |  | case study/ |
| Combining terms |  | or/16-21 |
|  |  | 6 and 11 and 14 |
|  |  | 23 not 22 |
| Final |  | remove duplicates from 24 |

**Supplementary Table S2: Full inclusion/exclusion criteria for studies in this review**

| Criteria | Inclusion | Exclusion |
| --- | --- | --- |
| Population | 1. Pregnant women requiring referral in an emergency or following an obstetric/pregnancy-related complication for childbirth care 2. Healthcare personnel including doctors, nurses, midwives, health/physician assistants targeted by relevant referral interventions 3. Community health workers, traditional birth attendants, community leaders or volunteers targeted by relevant referral interventions 4. Healthcare leaders or policy makers targeted by relevant referral interventions | 1. Referrals of new-borns 2. Pregnant women referred in non-obstetric emergency scenarios 3. Women with non-maternity related conditions |
| Intervention | Any intervention to:   1. Improve identification of obstetric emergencies that need to be referred by health professionals within the intrapartum or post-partum period (up to 42 days following delivery) 2. Improve communication of referrals by healthcare professionals in obstetric emergencies 3. Enable initiating facilities to inform receiving facilities about referral ahead of time 4. Enable feedback of the referral intervention and/or outcome by receiving facility to initiating facility 5. Improve communication of referrals from healthcare personnel or community health workers providing delivery services at homes   Single interventions and combinations of interventions aimed solely at improving communication and feedback of referral will be included.  Interventions must be critical to either phase two or phase three delays,(41) or a combination of the two, as by design it is often difficult to disentangle the interplay between components of an intervention in reducing these delays.(10)  Both single and two-way referral system interventions will be included.(42) | 1. Interventions to reduce phase one delays 2. Transport-only interventions 3. Interventions to only improve skills of health workers in managing obstetric complications that does not include identification of cases for referral 4. Interventions focused on reducing phase two delays that do not involve communication or feedback of referral 5. Interventions to improve non-emergency referrals |
| Comparison | Standard of care or groups without intervention | N/A |
| Outcome | Any outcome resulting from improved referral practice including but not limited to:  Output measures (proximal outcomes)   1. Travel time of referral between facilities 2. Cost associated with the referral process 3. Level of satisfaction of the referral process or outcome of referral intervention (for patient and/or health worker)   Intermediate measures   1. Rate of utilization of health facility services 2. Rate of compliance with referral process 3. Referral rates 4. Level of coordination between health facilities within an administrative district/region   Final outcomes   1. Absolute change in maternal mortality rate/ratio 2. Absolute change in neonatal mortality and stillbirth rates 3. Near-miss rates 4. Case fatality and complication rates   Outcomes should be measured from baseline were recorded and should be associated with the period of implementation of the relevant referral intervention. | 1. Neonatal deaths after the first week of life are often not related to complications of childbirth and would therefore be excluded |
| Setting | All countries in Sub-Saharan Africa as defined by the World Bank.(43)  This list includes: Angola, Benin, Botswana, Burkina Faso, Burundi, Cameroon, Cape Verde, Central African Republic, Chad, Comoros, Congo (Brazzaville), Congo (Democratic Republic), Cote d'Ivoire, Djibouti, Equatorial Guinea, Eritrea, Eswatini, Ethiopia, Gabon, Gambia, Ghana, Guinea, Guinea-Bissau, Kenya, Lesotho, Liberia, Madagascar, Malawi, Mali, Mauritania, Mauritius, Mozambique, Namibia, Niger, Nigeria, Rwanda, Sao Tome, Senegal, Seychelles, Sierra Leone, Somalia, South Africa or South Sudan, Sudan, Swaziland, Tanzania, Togo, Uganda, Zaire, Zambia and Zimbabwe. | Refugee camps, War Zones and Mass casualty scenarios  Studies set in any other countries |
| Research type | 1. Quantitative research 2. Qualitative research 3. Mixed-methods research | Any other non-primary research design |
| Study Design | 1. Quantitative research designs of emergency maternity referral systems from published and grey literature with a control or comparison group. This includes: 2. Randomized controlled trials 3. Quasi-experimental studies 4. Observational studies 5. Non-randomized prospective studies 6. Controlled before-after studies 7. Interrupted time series 8. Qualitative research methods with or without a comparison group will include: 9. Interviews 10. Focus group discussions 11. Key informant interviews   Methods should focus on relevant referral interventions | 1. Quantitative research designs without a comparison group 2. Cross-sectional studies 3. Comments 4. Editorials 5. Case reports 6. Case study 7. Historical article |
| Timeline | Outcomes measured at least one month after implementation of intervention | Study outcomes measured without temporality |
| Language limitation | Only studies in English Language | Studies in any other language |

**Supplementary Table S3**: List of included studies

| Number | Citation |
| --- | --- |
| 1 | Banke-Thomas A, Maua J, Madaj B, et al. Perspectives of stakeholders on emergency obstetric care training in Kenya: a qualitative study. International Health 2020;12:11–18 |
| 2 | Henry EG, Thea DM, Hamer DH, et al. The impact of a multi-level maternal health programme on facility delivery and capacity for emergency obstetric care in Zambia. Glob Public Health 2017;13:1481–1494. |
| 3 | Mangwi Ayiasi R, Atuyambe LM, Kiguli J, et al. Use of mobile phone consultations during home visits by Community Health Workers for maternal and newborn care: community experiences from Masindi and Kiryandongo districts, Uganda. BMC Public Health 2015;15:560. |
| 4 | Sevene E, Sharma S, Munguambe K, et al. Community-level interventions for pre-eclampsia (CLIP) in Mozambique: A cluster randomised controlled trial. Pregnancy Hypertension 2020;21:96–105. |
| 5 | Amoakoh-Coleman M, Ansah E, Klipstein-Grobusch K, et al. Completeness of obstetric referral letters/notes from subdistrict to district level in three rural districts in Greater Accra region of Ghana: an implementation research using mixed methods. BMJ Open 2019;9:e029785 |
| 6 | Amoakoh HB, Klipstein-Grobusch K, Ansah EK, et al. How and why front-line health workers (did not) use a multifaceted mHealth intervention to support maternal and neonatal healthcare decision-making in Ghana. BMJ Global Health 2019;4:e001153. |
| 7 | Dillip A, Kimatta S, Embrey M, et al. Can formalizing links among community health workers, accredited drug dispensing outlet dispensers, and health facility staff increase their collaboration to improve prompt access to maternal and child care? A qualitative study in Tanzania. BMC Health Services Research 2017;17:416. |
| 8 | Jacobs C, Michelo C, Moshabela M. Implementation of a community-based intervention in the most rural and remote districts of Zambia: a process evaluation of safe motherhood action groups. Implementation Science 2018;13:74. |
| 9 | Kanyesigye H, Muhwezi O, Kazungu C, et al. Will district health centres use preloaded cell phones for pre-referral phone calls for women in labour: a randomized pilot study at Mbarara Regional Referral Hospital in southwest Uganda. Can J Public Health 2019;110:520–522. |
| 10 | Ruton H, Musabyimana A, Grépin K, et al. Evaluating the Impact of RapidSMS: Final Report. :98. |
| 11 | Santos N, Mulowooza J, Isabirye N, et al. Effect of a labor triage checklist and ultrasound on obstetric referral at three primary health centers in Eastern Uganda. International Journal of Gynecology & Obstetrics n/a. Available at: https://obgyn.onlinelibrary.wiley.com/doi/abs/10.1002/ijgo.13420 [Accessed January 16, 2021]. |
| 12 | Ruton H, Musabyimana A, Gaju E, et al. The impact of an mHealth monitoring system on health care utilization by mothers and children: an evaluation using routine health information in Rwanda. Health Policy Plan 2018;33:920–927. |
| 13 | Akpala CO. An evaluation of the knowledge and practices of trained traditional birth attendants in Bodinga, Sokoto State, Nigeria. J Trop Med Hyg 1994;97:46–50. |
| 14 | Leigh B. The Use of Partograms by Maternal and Child Health Aides. Journal of Tropical Pediatrics 1986;32:107–110. |
| 15 | Mucunguzi S, Wamani H, Lochoro P, et al. Effects of Improved Access to Transportation on Emergency obstetric Care Outcomes in Uganda. :8. |

**Supplementary Table S4a**: List of studies excluded at full text stage based on language (English language), study design, studies restricted to humans only and studies in sub-Saharan Africa.

| **Author** | **Year** | **Title** |
| --- | --- | --- |
| Abauleth 2006 | 2006 | Prognosis of uterine rupture during labor: A 293-case series compiled at the Bouake University Hospital Center (Ivory Coast). French |
| Adolphson 2016 | 2016 | Midwives' experiences of working conditions, perceptions of professional role and attitudes towards mothers in Mozambique |
| Agbo 2013 | 2013 | TRAINING OF TRADITIONAL BIRTH ATTENDANTS: A STRATEGY FOR REDUCTION OF MATERNAL AND INFANT MORTALITY |
| Ameh 2016 | 2016 | Knowledge and skills of healthcare providers in sub-Saharan Africa and Asia before and after competency-based training in emergency obstetric and early Newborn Care |
| Ameh 2012 | 2012 | The impact of emergency obstetric care training in Somaliland, Somalia |
| Ameyaw 2020 | 2020 | Quality and women's satisfaction with maternal referral practices in sub-Saharan African low and lower-middle income countries: a systematic review |
| Anonymous 2019 | 2019 | Abstracts from Women's Health 2019 |
| Aradeon 2016 | 2016 | Reducing rural maternal mortality and the equity gap in northern Nigeria: The public health evidence for the community communication emergency referral strategy |
| Ariba 2006 | 2006 | Biosocial characteristics and mode of delivery at term of women monitored in a voluntary agency hospital |
| Awoonor-Williams 2015 | 2015 | Conducting an audit to improve the facilitation of emergency maternal and newborn referral in northern Ghana |
| Bailey 2011 | 2011 | Using a GIS to model interventions to strengthen the emergency referral system for maternal and newborn health in Ethiopia |
| Banke-Thomas 2020 | 2020 | Reaching health facilities in situations of emergency: qualitative study capturing experiences of pregnant women in Africa's largest megacity |
| Banke-Thomas 2019 | 2019 | Assessing geographical distribution and accessibility of emergency obstetric care in sub-Saharan Africa: a systematic review |
| Banke-Thomas 2016 | 2016 | Assessing emergency obstetric care provision in low- and middle-income countries: a systematic review of the application of global guidelines |
| Bazant 2013 | 2013 | Quality of care to prevent and treat postpartum hemorrhage and pre-eclampsia/ eclampsia: An observational assessment in Madagascar's hospitals |
| Belda 2016 | 2016 | Birth preparedness, complication readiness and other determinants of place of delivery among mothers in Goba District, Bale Zone, South East Ethiopia |
| Benson 2019 | 2019 | Assessment of maternal referral systems used for a rural Zambian hospital: the development of setting specific protocols for the identification of complications |
| Boatin 2017 | 2017 | Evaluating Facility-Based Decision-Making in Women with a Prior Cesarean Delivery and Association with Maternal and Perinatal Outcomes |
| Brenner 2015 | 2015 | The quality of clinical maternal and neonatal healthcare a strategy for identifying 'routine care signal functions' |
| Broccoli 2016 | 2016 | Community-based perceptions of emergency care in Zambian communities lacking formalised emergency medicine systems |
| Chen 2011 | 2011 | The effectiveness of continuing training for traditional birth attendants on their reproductive health-care knowledge and performance |
| Cranmer 2018 | 2018 | Beyond signal functions in global obstetric care: Using a clinical cascade to measure emergency obstetric readiness |
| Diallo 2020 | 2020 | Maternal mortality risk indicators: Case-control study at a referral hospital in Guinea |
| Dogba 2012 | 2012 | Qualification of staff, organization of services, and management of pregnant women in rural settings: The case of diema and kayes districts (Mali) |
| Donat 2009 | 2009 | Czech model for decrease of maternal mortality in Uganda. Czech |
| Dumont 2002 | 2002 | Maternal morbidity and qualification of health-care workers: Comparison between two different populations in Senegal. French |
| Eades 1993 | 1993 | Traditional birth attendants and maternal mortality in Ghana |
| Egbe 2020 | 2020 | Stillbirth rates and associated risk factors at the Buea and Limbe regional hospitals, Cameroon: A case-control study |
| Ellis 2011 | 2011 | Reducing maternal mortality in Uganda: applying the 'three delays' framework |
| Elmusharaf 2017 | 2017 | Patterns and determinants of pathways to reach comprehensive emergency obstetric and neonatal care (CEmONC) in South Sudan: Qualitative diagrammatic pathway analysis |
| Esienumoh 2018 | 2018 | Empowering members of a rural southern community in Nigeria to plan to take action to prevent maternal mortality: A participatory action research project |
| Fernandes 2016 | 2016 | NAMIBIAN MIDWIVES' EXPERIENCES OF USING THE PARTOGRAPH ON LABOURING WOMEN IN A REGIONAL HOSPITAL |
| Fisk 1989 | 1989 | Labor outcome of juvenile primiparae in a population with a high incidence of contracted pelvis |
| Floyd 2018 | 2018 | The introduction of a midwife-led obstetric triage system into a regional referral hospital in Ghana |
| Forshaw 2016 | 2016 | Exploring the third delay: an audit evaluating obstetric triage at Mulago National Referral Hospital |
| Gabrysch 2019 | 2019 | Does facility birth reduce maternal and perinatal mortality in Brong Ahafo, Ghana? A secondary analysis using data on 119Ã¢â‚¬Ë†244 pregnancies from two cluster-randomised controlled trials |
| Gebhardt 2015 | 2015 | Preparing for caesarean delivery |
| Gebhardt 2007 | 2007 | Standardised maternal guideline on the management of postpartum haemorrhage |
| Geelhoed 2018 | 2018 | Improving emergency obstetric care and reversing the underutilisation of vacuum extraction: A qualitative study of implementation in Tete Province, Mozambique |
| Geleto 2018 | 2018 | Barriers to access and utilization of emergency obstetric care at health facilities in sub-Saharan Africa-a systematic review protocol |
| Gidey 2013 | 2013 | Patterns of maternal mortality and associated factors; a case-control study at public hospitals in tigray region, ethiopia, 2012 |
| Goldenberg 2018 | 2018 | Routine antenatal ultrasound in low- and middle-income countries: first look - a cluster randomised trial |
| Goodman 2017 | 2017 | The third delay: Understanding waiting time for obstetric referrals at a large regional hospital in Ghana |
| Gunawardena 2018 | 2018 | Facility-Based Maternal Death in Western Africa: A Systematic Review |
| Heemelaar 2020 | 2020 | Maternal near-miss surveillance, Namibia |
| Horner 2014 | 2014 | Profile of patients and referrals at a midwife obstetric unit in Tshwane North subdistrict, Gauteng province |
| Houngnihin 2017 | 2017 | Understanding failure of obstetric referral to the Cotonou University gynaecology and obstetrics clinic |
| Hughes 2020 | 2020 | Decision-to-delivery interval of emergency cesarean section in Uganda: a retrospective cohort study |
| Hussein 2016 | 2016 | Maternal death and obstetric care audits in Nigeria: A systematic review of barriers and enabling factors in the provision of emergency care |
| Hussein 2012 | 2012 | The effectiveness of emergency obstetric referral interventions in developing country settings: A systematic review |
| Ifenne 1997 | 1997 | Improving the quality of obstetric care at the teaching hospital, Zaria, Nigeria |
| Irani 2015 | 2015 | Challenges affecting access to cesarean delivery and strategies to overcome them in low-income countries |
| De Brouwere, Vincent 2008 | 2008 | Do facility-based audits help West African hospitals to provide better care for patients with obstetric emergencies? |
| Florence Mgawadere 2018 | 2018 | Improving the quality of maternal and newborn health services in high priority districts in Malawi |
| Jackson 2016 | 2016 | Health extension workers' and mothers' attitudes to maternal health service utilization and acceptance in Adwa Woreda, Tigray Region, Ethiopia |
| John 2002 | 2002 | Knowledge and practice of traditional birth attendants concerning risk factors in pregnancy, labour and puerperium |
| Jonas 2017 | 2017 | Healthcare workers' behaviors and personal determinants associated with providing adequate sexual and reproductive healthcare services in sub-Saharan Africa: A systematic review |
| Kalisa 2016 | 2016 | Maternal Near Miss and quality of care in a rural Rwandan hospital |
| Kamath-Rayne 2015 | 2015 | Resuscitation and Obstetrical Care to Reduce Intrapartum-Related Neonatal Deaths: A MANDATE Study |
| Kandeh 1997 | 1997 | Community motivators promote use of emergency obstetric services in rural Sierra Leone |
| Kasahun 2018 | 2018 | Predictors of maternal near miss among women admitted in Gurage zone hospitals, South Ethiopia, 2017: A case control study |
| Kaye 2003 | 2003 | Antenatal and intrapartum risk factors for birth asphyxia among emergency obstetric referrals in Mulago Hospital, Kampala, Uganda |
| Ketema 2020 | 2020 | Effects of maternal education on birth preparedness and complication readiness among Ethiopian pregnant women: A systematic review and meta-analysis |
| Keyes 2019 | 2019 | Geographic access to emergency obstetric services: a model incorporating patient bypassing using data from Mozambique |
| Kinenkinda 2017 | 2017 | Risk factors for maternal and perinatal mortality among women undergoing cesarean section in Lubumbashi, Democratic Republic of Congo II. French |
| Kongnyuy 2008 | 2008 | Criteria-based audit to improve a district referral system in Malawi: A pilot study |
| Kumela 2020 | 2020 | Determinants of Maternal Near Miss in Western Ethiopia |
| Le Bacq 1997 | 1997 | High maternal mortality levels and additional risk from poor accessibility in two districts of Northern Province, Zambia |
| Liambila 2013 | 2013 | The community midwifery model in Kenya: Expanding access to comprehensive reproductive health services at the community level |
| Lindtjorn 2018 | 2018 | Reducing stillbirths in Ethiopia: Results of an intervention programme |
| Lindtjorn 2017 | 2017 | Reducing maternal deaths in Ethiopia: Results of an intervention programme in Southwest Ethiopia |
| Lobis 2011 | 2011 | Expected to deliver: Alignment of regulation, training, and actual performance of emergency obstetric care providers in Malawi and Tanzania |
| Lori 2010 | 2010 | Home-Based Life-Saving Skills in Liberia: Acquisition and Retention of Skills and Knowledge |
| Luck 2000 | 2000 | Safe motherhood intervention studies in Africa: a review |
| Luhete 2017 | 2017 | Study of maternal and perinatal prognosis for vaginal delivery in adolescent girls in Lubumbashi, democratic republic of the Congo. French |
| Lungu 2007 | 2007 | Does the upgrading of the radio communications network in health facilities reduce the delay in the referral of obstetric emergencies in Southern Malawi? |
| Maine 1997 | 1997 | Lessons for program design from the PMM projects |
| Mburu 2018 | 2018 | A model for predicting utilization of mHealth interventions in low-resource settings: case of maternal and newborn care in Kenya |
| Meda 2008 | 2008 | From evaluating a Skilled Care Initiative in rural Burkina Faso to policy implications for safe motherhood in Africa |
| Meda 2011 | 2011 | Leadership and vision in the improvement of universal health care coverage in low-income countries |
| Monteiro 2016 | 2016 | Obstetric emergency training in a rural South African hospital: simple measures improve knowledge |
| Morof 2019 | 2019 | Addressing the Third Delay in Saving Mothers, Giving Life Districts in Uganda and Zambia: Ensuring Adequate and Appropriate Facility-Based Maternal and Perinatal Health Care |
| Nathan 2018 | 2018 | The CRADLE vital signs alert: qualitative evaluation of a novel device designed for use in pregnancy by healthcare workers in low-resource settings |
| Peter von Dadelszen 2020 | 2013 | Community Level Interventions for Pre-eclampsia |
| Ndiaye 2001 | 2001 | A new decision-making tool in the fight against maternal mortality: Dystocial risk score (DRS). French |
| Ndiaye 2013 | 2013 | Dystocia risk score: a decision making tool to combat maternal mortality |
| Ngabo 2012 | 2012 | Designing and Implementing an Innovative SMS-based alert system (RapidSMS-MCH) to monitor pregnancy and reduce maternal and child deaths in Rwanda |
| Ngoma 2019 | 2019 | Addressing the Second Delay in Saving Mothers, Giving Life Districts in Uganda and Zambia: Reaching Appropriate Maternal Care in a Timely Manner |
| Ni Bhuinneain 2015 | 2015 | A systematic review of essential obstetric and newborn care capacity building in rural sub-Saharan Africa |
| Nwakoby 1997 | 1997 | Community contact persons promote utilization of obstetric services, Anambra State, Nigeria |
| Nyamtema 2017 | 2017 | Introducing eHealth strategies to enhance maternal and perinatal health care in rural Tanzania |
| Olukoya 1997 | 1997 | Upgrading obstetric care at a secondary referral hospital, Ogun State, Nigeria |
| Owen 2019 | 2019 | Use of a cross-platform messaging technology to strengthen the obstetric referral system in the Greater Accra region, Ghana: findings from a pilot programme |
| Mugyenyi 2017 | 2020 | ASSESSMENT OF INTRAPARTUM REFERRALS AND USE OF MOBILE PHONE TECHNOLOGY TO IMPROVE MATERNAL-FETAL OUTCOMES IN SOUTH WESTERN UGANDA |
| Pattinson 2019 | 2019 | Reducing maternal deaths by skills-and-drills training in managing obstetric emergencies: A before-and-after observational study |
| Pattinson 2018 | 2018 | Obstetrics knowledge and skills training as a catalyst for change |
| Ramaswamy 2015 | 2015 | Transforming Maternal and Neonatal Outcomes in Tertiary Hospitals in Ghana: An Integrated Approach for Systems Change |
| Sabitu 1997 | 1997 | The effect of improving maternity services in a secondary facility, Zaria, Nigeria |
| Salgado 2017 | 2017 | A service concept and tools to improve maternal and newborn health in Nigeria and Uganda |
| Samai 1997 | 1997 | Facilitating emergency obstetric care through transportation and communication, Bo, Sierra Leone |
| Santos 2006 | 2006 | Improving emergency obstetric care in Mozambique: The story of Sofala |
| Shimoda 2015 | 2015 | Midwives' intrapartum monitoring process and management resulting in emergency referrals in Tanzania: A qualitative study |
| Sibley 2004 | 2004 | The American College of Nurse-Midwives' home-based lifesaving skills program: A review of the Ethiopia field test |
| Sotunsa 2016 | 2016 | Community health workers' knowledge and ' practice in relation to pre-eclampsia in Ogun State, Nigeria: an essential bridge to maternal survival |
| Titilayo 2014 | 2014 | Assessment of Mobile Health Nursing Intervention Knowledge among Community Health Nurses in Oyo State, Nigeria |
| Vinayak 2017 | 2017 | Training Midwives to Perform Basic Obstetric Point-of-Care Ultrasound in Rural Areas Using a Tablet Platform and Mobile Phone Transmission Technology-A WFUMB COE Project |
| Vousden 2018 | 2018 | Evaluation of a novel vital sign device to reduce maternal mortality and morbidity in low-resource settings: a mixed method feasibility study for the CRADLE-3 trial |
| Wekesah 2016 | 2016 | Effective non-drug interventions for improving outcomes and quality of maternal health care in sub-Saharan Africa: A systematic review |

**Supplementary Table S4b**: List of studies excluded at full text stage based on population (pregnant women requiring referral due to obstetric emergencies, healthcare personnel involved in the referral process)

| **Author** | **Year** | **Title** |
| --- | --- | --- |
| Afulani 2019 | 2019 | Can integrated clinical simulation trainings improve person-centred maternity care? Results from a pilot project in Ghana |
| Ajeani 2017 | 2017 | A cascade model of mentorship for frontline health workers in rural health facilities in Eastern Uganda: processes, achievements and lessons |
| Ameh 2018 | 2018 | Retention of knowledge and skills after Emergency Obstetric Care training: A multi-country longitudinal study |
| Amoakoh-Coleman 2020 | 2020 | Coverage of intermittent preventive treatment of malaria in pregnancy (IPTp) influences delivery outcomes among women with obstetric referrals at the district level in Ghana |
| Ballard 2013 | 2013 | Provision of individualised obstetric risk advice to increase health facility usage by women at risk of a complicated delivery: A cohort study of women in the rural highlands of West Ethiopia |
| Benski 2017 | 2017 | Usability and feasibility of a mobile health system to provide comprehensive antenatal care in low-income countries: PANDA mHealth pilot study in Madagascar |
| Bowser 2018 | 2018 | Cost effectiveness of mobile health for antenatal care and facility births in Nigeria |
| Callaghan-Koru 2013 | 2013 | Contribution of community-based newborn health promotion to reducing inequities in healthy newborn care practices and knowledge: evidence of improvement from a three-district pilot program in Malawi |
| Steege R 2018 | 2018 | 'The phone is my boss and my helper' â€“ A gender analysis of an mHealth intervention with Health Extension Workers in Southern Ethiopia |
| Cofie 2018 | 2018 | A qualitative study of women's network social support and facility delivery in rural Ghana |
| Cohen 2017 | 2017 | Measuring The Impact Of Cash Transfers And Behavioral 'Nudges' On Maternity Care In Nairobi, Kenya |
| Coleman 2020 | 2020 | Evaluating the effect of maternal mHealth text messages on uptake of maternal and child health care services in South Africa: a multicentre cohort intervention study |
| Coleman 2020 | 2020 | The Mobile Alliance for Maternal Action Text Message-Based mHealth Intervention for Maternal Care in South Africa: Qualitative User Study |
| De Brouwere 2009 | 2009 | Task shifting for emergency obstetric surgery in district hospitals in Senegal |
| Dettinger 2018 | 2018 | Measuring movement towards improved emergency obstetric care in rural Kenya with implementation of the PRONTO simulation and team training program |
| Dongmo 2006 | 2006 | Introduction of an obstetric health information system: Results of a pilot study in North Cameroon |
| Ensor 2014 | 2014 | Mobilizing communities to improve maternal health: Results of an intervention in rural Zambia |
| Evjen-Olsen 2009 | 2009 | Achieving progress in maternal and neonatal health through integrated and comprehensive healthcare services - Experiences from a programme in northern Tanzania |
| Eze 2020 | 2020 | Unlocking community capabilities for addressing social norms/practices: Behavioural change intervention study to improve birth preparedness and complication readiness among pregnant women in rural Nigeria |
| Fofana 1997 | 1997 | Promoting the use of obstetric services through community loan funds, Bo, Sierra Leone |
| Foord 1995 | 1995 | Gambia: evaluation of the mobile health care service in West Kiang district |
| Fox-Rushby 1995 | 1995 | The Gambia: cost and effectiveness of a mobile maternal health care service, West Kiang |
| Fox-Rushby 1996 | 1996 | Costs, effects and cost-effectiveness analysis of a mobile maternal health care service in West Kiang, The Gambia |
| Fujita 2012 | 2012 | Humanised care and a change in practice in a hospital in Benin |
| Ganaba 2016 | 2016 | The obstetric care subsidy policy in Burkina Faso: what are the effects after five years of implementation? Findings of a complex evaluation |
| Geerts 1996 | 1996 | Routine obstetric ultrasound examinations in South Africa: Cost and effect on perinatal outcome - A prospective randomised controlled trial |
| Greenwold 2014 | 2014 | Implementing an obstetric ultrasound training program in rural Africa |
| Gummi 1997 | 1997 | Community education to encourage use of emergency obstetric services, Kebbi State, Nigeria. The Sokoto PMM Team |
| Hackett 2018 | 2018 | Impact of smartphone-assisted prenatal home visits on women’s use of facility delivery: results from a cluster-randomized trial in rural Tanzania |
| Hirai 2020 | 2020 | The impact of supply-side and demand-side interventions on use of antenatal and maternal services in western Kenya: A qualitative study |
| Homaifar 2013 | 2013 | Emergency obstetrics knowledge and practical skills retention among medical students in Rwanda following a short training course |
| Jackson 2017 | 2017 | Factors that hinder or enable maternal health strategies to reduce delays in rural and pastoralist areas in Ethiopia |
| Jha 1998 | 1998 | The cost-effectiveness of forty health interventions in Guinea |
| Kayongo 2006 | 2006 | Improving availability of EmOC services in Rwanda - CARE's experiences and lessons learned at Kabgayi Referral Hospital |
| Kayongo 2006 | 2006 | Making EmOC a reality - CARE's experiences in areas of high maternal mortality in Africa |
| Kermode 2017 | 2017 | Walking Together: Towards a Collaborative Model for Maternal Health Care in Pastoralist Communities of Laikipia and Samburu, Kenya |
| Keyes 2011 | 2011 | Ethiopia's assessment of emergency obstetric and newborn care: Setting the gold standard for national facility-based assessments |
| Kruk 2016 | 2016 | Evaluation Of a Maternal Health Program In Uganda And Zambia Finds Mixed Results On Quality Of Care And Satisfaction |
| Laar 2019 | 2019 | Assessment of mobile health technology for maternal and child health services in rural Upper West Region of Ghana |
| Little 2013 | 2013 | Meeting community health worker needs for maternal health care service delivery using appropriate mobile technologies in Ethiopia |
| Lombaard 2005 | 2005 | Evaluation of a strict protocol approach in managing women with severe disease due to hypertension in pregnancy: A before and after study |
| Lund 2014 | 2014 | Mobile phones improve antenatal care attendance in Zanzibar: A cluster randomized controlled trial |
| Maaloe 2018 | 2018 | Effect of locally tailored labour management guidelines on intrahospital stillbirths and birth asphyxia at the referral hospital of Zanzibar: a quasi-experimental pre-post study (The PartoMa study) |
| MacDonald 2019 | 2019 | Socio-cultural contextual factors that contribute to the uptake of a mobile health intervention to enhance maternal health care in rural Senegal |
| Marsh 2011 | 2011 | Training birth attendants in rural Zambia in neonatal resuscitation, and the use of amoxicillin coupled with facilitated referral, reduces neonatal mortality |
| Mbachu 2018 | 2018 | Tracking stillbirths by referral pattern and causes in a rural tertiary hospital in Southern Nigeria |
| Mbonye 2007 | 2007 | Emergency obstetric care as the priority intervention to reduce maternal mortality in Uganda |
| McCarthy 2015 | 2015 | A feasible, acceptable and effective way to teach health care workers in low- and middle-income countries a method to manage acutely ill obstetric women |
| McClure 2014 | 2014 | First look: a cluster-randomized trial of ultrasound to improve pregnancy outcomes in low income country settings |
| McConnell 2016 | 2016 | Can a community health worker administered postnatal checklist increase health-seeking behaviors and knowledge?: evidence from a randomized trial with a private maternity facility in Kiambu County, Kenya |
| Murray 2001 | 2001 | Tools for monitoring the effectiveness of district maternity referral systems |
| Mwase 2020 | 2020 | Experiences of using the toll-free telephone line to access maternal and newborn health services in central Uganda: a qualitative study |
| McClure 2014 | 2013 | A Cluster-Randomized Trial of Ultrasound Use to Improve Pregnancy Outcomes in Low Income Country Settings |
| Lund 2014 | 2013 | Wired Mothers - Use of Mobile Phones to Improve Maternal and Neonatal Health in Zanzibar |
| Ndiaye 2001 | 2001 | A new decision support tool in the campaign against maternal mortality: the Dystocia Risk Score |
| Nyamtema 2016 | 2016 | Enhancing maternal and perinatal health in under-served remote areas in sub-Saharan Africa: A Tanzanian model |
| Okonofua 2020 | 2020 | Outcome of multifaceted interventions for improving the quality of antenatal care in Nigerian referral hospitals |
| Oladapo 2009 | 2009 | National data system on near miss and maternal death: Shifting from maternal risk to public health impact in Nigeria |
| Ratcliffe 2016 | 2016 | Mitigating disrespect and abuse during childbirth in Tanzania: An exploratory study of the effects of two facility-based interventions in a large public hospital |
| Rosenberg 2020 | 2020 | Prehospital Emergency Obstetric and Neonatal Care Train the Trainers program in Rwanda |
| Schmitz 2019 | 2019 | Did Saving Mothers, Giving Life Expand Timely Access to Lifesaving Care in Uganda? A Spatial District-Level Analysis of Travel Time to Emergency Obstetric and Newborn Care |
| Sialubanje 2017 | 2017 | Increasing utilisation of skilled facility-based maternal healthcare services in rural Zambia: The role of safe motherhood action groups |
| Tang 2016 | 2016 | Improvement and retention of emergency obstetrics and neonatal care knowledge and skills in a hospital mentorship program in Lilongwe, Malawi |
| Tiruneh 2018 | 2018 | The effect of implementation strength of basic emergency obstetric and newborn care (BEmONC) on facility deliveries and the met need for BEmONC at the primary health care level in Ethiopia |
| Yigzaw 2019 | 2019 | Comparing the effectiveness of a blended learning approach with a conventional learning approach for basic emergency obstetric and newborn care training in Ethiopia |
| de Ramirez 2014 | 2014 | Emergency response in resource-poor settings: a review of a newly-implemented EMS system in rural Uganda |

**Supplementary Table S4c**: List of studies excluded at full text stage based on the relevant type of referral intervention (emergency obstetric referral decision making, communication and feedback)

| **Author** | **Year** | **Title** |
| --- | --- | --- |
| Nyamtema 2016 | 2016 | Increasing the availability and quality of caesarean section in Tanzania |
| Abraham 2014 | 2014 | Predictors of labor abnormalities in university hospital: Unmatched case control study |
| Accorsi 2017 | 2017 | Cost-effectiveness of an ambulance-based referral system for emergency obstetrical and neonatal care in rural Ethiopia |
| Afari 2014 | 2014 | Can an integrated obstetric emergency simulation training improve respectful maternity care? Results from a pilot study in Ghana |
| Ahluwalia 2010 | 2010 | An evaluation of a community-based approach to safe motherhood in northwestern Tanzania |
| Ahluwalia 2012 | 2012 | Post-Project Assessment of Community-supported Emergency Transport Systems for Health Care Services in Tanzania |
| Ahluwalia 2003 | 2003 | An evaluation of a community-based approach to safe motherhood in northwestern Tanzania |
| Alwy Al-Beity 2020 | 2020 | We do what we can do to save a woman health workers' perceptions of health facility readiness for management of postpartum haemorrhage |
| Amoakoh 2017 | 2017 | The effect of a clinical decision-making mHealth support system on maternal and neonatal mortality and morbidity in Ghana |
| Amoakoh 2019 | 2019 | Using Mobile Health to Support Clinical Decision-Making to Improve Maternal and Neonatal Health Outcomes in Ghana: Insights of Frontline Health Worker Information Needs |
| Awoonor-Williams 2013 | 2013 | The Ghana essential health interventions program: a plausibility trial of the impact of health systems strengthening on maternal & child survival |
| Bagayoko 2017 | 2017 | The delegation of tasks in the era of e-health to support community interventions in maternal and child health: lessons learned from the PACT-Denbaya project |
| Bang 2018 | 2018 | Effects of a Community Outreach Program for Maternal Health and Family Planning in Tigray, Ethiopia |
| Banke-Thomas 2019 | 2019 | Social return on investment of emergency obstetric care training in Kenya |
| Bhopal 2013 | 2013 | Emergency obstetric referral in rural Sierra Leone: what can motorbike ambulances contribute? A mixed-methods study |
| Bolan 2018 | 2018 | mLearning in the Democratic Republic of the Congo: A Mixed-Methods Feasibility and Pilot Cluster Randomized Trial Using the Safe Delivery App |
| Bonawitz 2019 | 2019 | Quality and utilization patterns of maternity waiting homes at referral facilities in rural Zambia: A mixed-methods multiple case analysis of intervention and standard of care sites |
| Brazier 2014 | 2014 | Rethinking how to promote maternity care-seeking: factors associated with institutional delivery in Guinea |
| Brenner 2014 | 2014 | Design of an impact evaluation using a mixed methods model--an explanatory assessment of the effects of results-based financing mechanisms on maternal healthcare services in Malawi |
| Browning 2015 | 2015 | Reducing maternal morbidity and mortality in the developing world: A simple, cost-effective example |
| Cole-Ceesay 2010 | 2010 | Strengthening the emergency healthcare system for mothers and children in the Gambia |
| Dehne 1995 | 1995 | Training birth attendants in the Sahel |
| Des Lauriers 2019 | 2019 | Putting the three delays model to work: A pragmatic 12-month community-based cohort study to assess access to emergency obstetrical and neonatal care in a remote island community in Western Kenya |
| DesLauriers 2020 | 2020 | The MOMENTUM study: Putting the 'Three Delays' to work to evaluate access to emergency obstetric and neonatal care in a remote island community in Western Kenya |
| Dickinson 2019 | 2019 | Improving the quality of midwifery training in resource limited settings: A quasi-experimental study |
| Drost 2010 | 2010 | Implementing safe motherhood: a low-cost intervention to improve the management of eclampsia in a referral hospital in Malawi |
| Duke 1997 | 1997 | Collaboration with government leaders to promote sustainability, Cross River State, Nigeria |
| Dumont 2013 | 2013 | Quality of care, risk management, and technology in obstetrics to reduce hospital-based maternal mortality in Senegal and Mali (QUARITE): A cluster-randomised trial |
| Dumont 2009 | 2009 | QUARITE (quality of care, risk management and technology in obstetrics): A cluster-randomized trial of a multifaceted intervention to improve emergency obstetric care in Senegal and Mali |
| Dumont 2005 | 2005 | Emergency obstetric care in developing countries: Impact of guidelines implementation in a community hospital in Senegal |
| Egenberg 2017 | 2017 | Impact of multi-professional, scenario-based training on postpartum hemorrhage in Tanzania: A quasi-experimental, pre- vs. post-intervention study |
| Ellard 2014 | 2014 | Can training in advanced clinical skills in obstetrics, neonatal care and leadership, of non-physician clinicians in Malawi impact on clinical services improvements (the ETATMBA project): A process evaluation |
| Ellard 2016 | 2016 | Up-skilling associate clinicians in Malawi in emergency obstetric, neonatal care and clinical leadership: the ETATMBA cluster randomised controlled trial |
| Ellard 2016 | 2016 | Can training non-physician clinicians/associate clinicians (NPCs/ACs) in emergency obstetric, neonatal care and clinical leadership make a difference to practice and help towards reductions in maternal and neonatal mortality in rural Tanzania? The ETATMBA project |
| Essien 1997 | 1997 | Community loan funds and transport services for obstetric emergencies in northern Nigeria |
| Ferguson 2020 | 2020 | Results-based financing to increase uptake of skilled delivery services in The Gambia: using the 'three delays' model to interpret midline evaluation findings |
| Filippi 2004 | 2004 | Obstetric audit in resource-poor settings: Lessons from a multi-country project auditing 'near miss' obstetrical emergencies |
| Fournier 2009 | 2009 | Improved access to comprehensive emergency obstetric care and its effect on institutional maternal mortality in rural Mali |
| Geerts 2004 | 2004 | A community-based obstetric ultrasound service |
| Gelano 2018 | 2018 | Effect of Mobile-health on maternal health care service utilization in Eastern Ethiopia: Study protocol for a randomized controlled trial |
| Gueye 2017 | 2017 | Simulation training for emergency obstetric and neonatal care in Senegal preliminary results |
| Habtamu 2019 | 2019 | Reducing maternal and neonatal mortality and morbidity by strengthening midwives' skills and improving their work setting in arsi zone, Oromia Region, Ethiopia-end-of-project evaluation report 2013-2016 |
| Hanson 2014 | 2014 | Expanded Quality Management Using Information Power (EQUIP): protocol for a quasi-experimental study to improve maternal and newborn health in Tanzania and Uganda |
| Hofman 2014 | 2014 | Experiences with facility-based maternal death reviews in northern Nigeria |
| Hofman 2008 | 2008 | Motorcycle ambulances for referral of obstetric emergencies in rural Malawi: Do they reduce delay and what do they cost? |
| Igwegbe 2012 | 2012 | Improving maternal mortality at a university teaching hospital in Nnewi, Nigeria |
| van den Broek 2019 | 2016 | Making it Happen: reducing maternal and neonatal deaths in South Africa; healthcare facility level assessment of in-service training in emergency obstetric and early newborn care for staff working in maternity services in twelve districts in RSA |
| Ameh 2018 | 2017 | Assessment of the impact of mentorship/supportive supervision to support health care workers learning |
| Ameh 2017 | 2017 | Investigation of the effect of emergency obstetric care training interventions on the knowledge and skills of final year midwifery students in Kenya |
| Sam Harper 2018 | 2018 | Tabora Maternal and Newborn Health Initiative: improving reproductive, maternal and newborn health in Tabora, Tanzania |
| Kabo 2019 | 2019 | Strengthening and monitoring health system's capacity to improve availability, utilization and quality of emergency obstetric care in northern Nigeria |
| Kabore 2019 | 2019 | DECIDE: A cluster-randomized controlled trial to reduce unnecessary caesarean deliveries in Burkina Faso |
| Kabuya 2020 | 2020 | Impact of maternal death reviews at a rural hospital in Zambia: A mixed methods study |
| Kadia 2020 | 2020 | Evaluation of emergency obstetric and neonatal care services in Kumba Health District, Southwest region, Cameroon (2011-2014): A before-after study |
| Kaiser 2019 | 2019 | The effects of maternity waiting homes on the health workforce and maternal health service delivery in rural Zambia: a qualitative analysis |
| Kawooya 2015 | 2015 | Impact of introducing routine antenatal ultrasound services on reproductive health indicators in Mpigi District, Central Uganda |
| Kisakye 2017 | 2017 | Effect of support supervision on maternal and newborn health services and practices in Rural Eastern Uganda |
| Klokkenga 2019 | 2019 | The effect of smartphone training of Ghanaian midwives by the Safe Delivery application on the incidence of postpartum hemorrhage: A cluster randomised controlled trial |
| Laisser 2019 | 2019 | Crisis: an educational game to reduce mortality and morbidity |
| Lalonde 2003 | 2003 | The FIGO Save the Mothers Initiative: The Uganda-Canada collaboration |
| Lamont 2016 | 2016 | Short message service (SMS) as an educational tool during pregnancy: A literature review |
| Leigh 1997 | 1997 | Improving emergency obstetric care at a district hospital, Makeni, Sierra Leone |
| Lori 2012 | 2012 | Behavior Change Following Implementation of Home-Based Life-Saving Skills in Liberia, West Africa |
| Lynch 1994 | 1994 | The impact of training and supervision on traditional birth attendants |
| Majoko 2005 | 2005 | Effectiveness of referral system for antenatal and intra-partum problems in Gutu district, Zimbabwe |
| Mangwi Ayiasi 2016 | 2016 | Effect of Village Health Team Home Visits and Mobile Phone Consultations on Maternal and Newborn Care Practices in Masindi and Kiryandongo, Uganda: a Community-Intervention Trial |
| Masoi 2019 | 2019 | Improving pregnant women's knowledge on danger signs and birth preparedness practices using an interactive mobile messaging alert system in Dodoma region, Tanzania: a controlled quasi experimental study |
| Mbaruku 1995 | 1995 | Reducing maternal mortality in Kigoma, Tanzania |
| Mbaruku 2018 | 2018 | Implementation project of the non-pneumatic anti-shock garment and m-communication to enhance maternal health care in rural Tanzania |
| Muhumuza Kananura 2017 | 2017 | Effect of a participatory multisectoral maternal and newborn intervention on birth preparedness and knowledge of maternal and newborn danger signs among women in Eastern Uganda: a quasi-experiment study |
| Furaha 2016 | 2010 | Home Based Life Saving Skills Training in a Rural Area in Tanzania |
| Bergen 2019 | 2017 | An Implementation Study of Interventions to Promote Safe Motherhood in Jimma Zone Ethiopia |
| Diego Bassani 2018 | 2018 | Low-dose High-frequency Training of Facility Health Care Providers in Mali |
| Nelson 2012 | 2012 | Evaluation of a novel training package among frontline maternal, newborn, and child health workers in South Sudan |
| Neumann 1986 | 1986 | Evaluation of a programme to train traditional birth attendants in Ghana |
| Nyamtema 2011 | 2011 | Tanzanian lessons in using non-physician clinicians to scale up comprehensive emergency obstetric care in remote and rural areas |
| Oguntunde 2018 | 2018 | Emergency transport for obstetric emergencies: Integrating community-level demand creation activities for improved access to maternal, newborn, and child health services in northern nigeria |
| Onono 2019 | 2019 | Narratives of women using a 24-hour ride-hailing transport system to increase access and utilization of maternal and newborn health services in rural western Kenya: A qualitative study |
| Otchere 2007 | 2007 | The challenges of improving emergency obstetric care in two rural districts in Mali |
| Oyesola 1997 | 1997 | Improving emergency obstetric care at a state referral hospital, Kebbi State, Nigeria |
| Pandey 2018 | 2018 | Re: Effect of locally-tailored labour management guidelines on intrahospital stillbirths and birth asphyxia at the referral hospital of Zanzibar: a quasi-experimental pre-post-study (The PartoMa study) |
| Pasha 2010 | 2010 | Communities, birth attendants and health facilities: a continuum of emergency maternal and newborn care (the Global Network's EmONC trial) |
| Schoon 2013 | 2013 | Impact of inter-facility transport on maternal mortality in the Free State Province |
| Serbanescu 2019 | 2019 | Impact of the Saving Mothers, Giving Life Approach on Decreasing Maternal and Perinatal Deaths in Uganda and Zambia |
| Serbanescu 2017 | 2017 | Rapid reduction of maternal mortality in Uganda and Zambia through the saving mothers, giving life initiative: Results of year 1 evaluation |
| Shikuku 2019 | 2019 | Reducing intrapartum fetal deaths through low-dose high frequency clinical mentorship in a rural hospital in Western Kenya: A quasi-experimental study |
| Sibley 2006 | 2006 | Home-Based Life Saving Skills in Ethiopia: An Update on the Second Phase of Field Testing |
| Sorensen 2010 | 2010 | Impact of ALSO training on the management of prolonged labor and neonatal care at Kagera Regional Hospital, Tanzania |
| Spitzer 2014 | 2014 | One-year evaluation of the impact of an emergency obstetric and neonatal care training program in Western Kenya |
| Srofenyoh 2016 | 2016 | Measuring the impact of a quality improvement collaboration to decrease maternal mortality in a Ghanaian regional hospital |
| Thomsen 2019 | 2019 | Health workers' experiences with the Safe Delivery App in West Wollega Zone, Ethiopia: A qualitative study |
| Tomedi 2015 | 2015 | From home deliveries to health care facilities: establishing a traditional birth attendant referral program in Kenya |
| Van Tetering 2020 | 2020 | Study protocol training for life: A stepped wedge cluster randomized trial about emergency obstetric simulation-based training in a low-income country |
| Vousden 2016 | 2016 | Lessons learnt during the implementation of a novel vital sign device and training package across three low-resource settings: a mixed method feasibility study for the CRADLE trial |
| Webber 2019 | 2019 | Challenges and Successes of Distributing Birth Kits with Misoprostol to Reduce Maternal Mortality in Rural Tanzania |
| Webber 2020 | 2020 | Experiences of a multiple intervention trial to increase maternity care access in rural Tanzania: Focus group findings with women, nurses and community health workers |
| Willcox 2017 | 2017 | Incremental cost and cost-effectiveness of low-dose, high-frequency training in basic emergency obstetric and newborn care as compared to status quo: part of a cluster-randomized training intervention evaluation in Ghana |
| Williams 2020 | 2020 | A protocol for evaluating a multi-level implementation theory to scale-up obstetric triage in referral hospitals in Ghana |
| Xiong 2019 | 2019 | Improving the quality of maternal and newborn health outcomes through a clinical mentorship program in the Democratic Republic of the Congo: Study protocol |
| van den Broek 2019 | 2019 | Effects of emergency obstetric care training on maternal and perinatal outcomes: a stepped wedge cluster randomised trial in South Africa |

**Supplementary Table S4d**: List of excluded studies at full text stage based on outcomes of interest (rate of referrals, facility utilization, maternal and early neonatal mortality including stillbirths)

| **Author** | **Year** | **Title** |
| --- | --- | --- |
| Amoakoh 2017 | 2017 | The effect of a clinical decision-making mHealth support system on maternal and neonatal mortality and morbidity in Ghana |
| Awoonor-Williams 2013 | 2013 | The Ghana essential health interventions program: a plausibility trial of the impact of health systems strengthening on maternal & child survival |
| Banke-Thomas 2019 | 2019 | Social return on investment of emergency obstetric care training in Kenya |
| Cole-Ceesay 2010 | 2010 | Strengthening the emergency healthcare system for mothers and children in the Gambia |
| Des Lauriers 2019 | 2019 | Putting the three delays model to work: A pragmatic 12-month community-based cohort study to assess access to emergency obstetrical and neonatal care in a remote island community in Western Kenya |
| DesLauriers 2020 | 2020 | The MOMENTUM study: Putting the 'Three Delays' to work to evaluate access to emergency obstetric and neonatal care in a remote island community in Western Kenya |
| Dumont 2009 | 2009 | QUARITE (quality of care, risk management and technology in obstetrics): A cluster-randomized trial of a multifaceted intervention to improve emergency obstetric care in Senegal and Mali |
| Fournier 2009 | 2009 | Improved access to comprehensive emergency obstetric care and its effect on institutional maternal mortality in rural Mali |
| Gelano 2018 | 2018 | Effect of Mobile-health on maternal health care service utilization in Eastern Ethiopia: Study protocol for a randomized controlled trial |
| Hanson 2014 | 2014 | Expanded Quality Management Using Information Power (EQUIP): protocol for a quasi-experimental study to improve maternal and newborn health in Tanzania and Uganda |
| Sam Harper 2018 | 2018 | Tabora Maternal and Newborn Health Initiative: improving reproductive, maternal and newborn health in Tabora, Tanzania |
| Lori 2012 | 2012 | Behavior Change Following Implementation of Home-Based Life-Saving Skills in Liberia, West Africa |
| Diego Bassani 2018 | 2018 | Low-dose High-frequency Training of Facility Health Care Providers in Mali |
| Pasha 2010 | 2010 | Communities, birth attendants and health facilities: a continuum of emergency maternal and newborn care (the Global Network's EmONC trial) |
| Srofenyoh 2016 | 2016 | Measuring the impact of a quality improvement collaboration to decrease maternal mortality in a Ghanaian regional hospital |
| Van Tetering 2020 | 2020 | Study protocol training for life: A stepped wedge cluster randomized trial about emergency obstetric simulation-based training in a low-income country |
| Williams 2020 | 2020 | A protocol for evaluating a multi-level implementation theory to scale-up obstetric triage in referral hospitals in Ghana |
| Xiong 2019 | 2019 | Improving the quality of maternal and newborn health outcomes through a clinical mentorship program in the Democratic Republic of the Congo: Study protocol |

**Supplementary Figure S1: Detailed PRISMA chart**

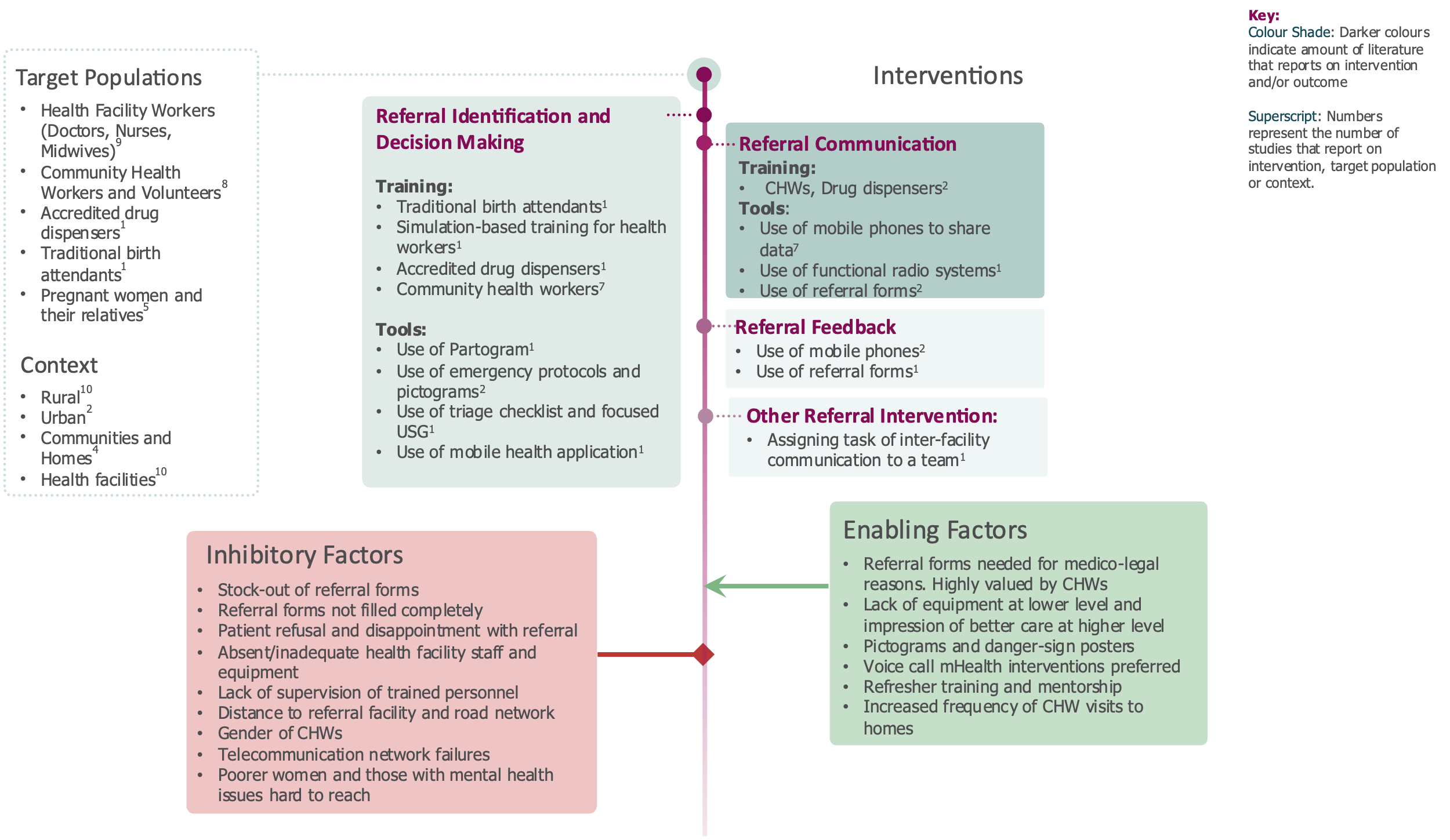
Supplementary Figure S2: Theory of Change Model


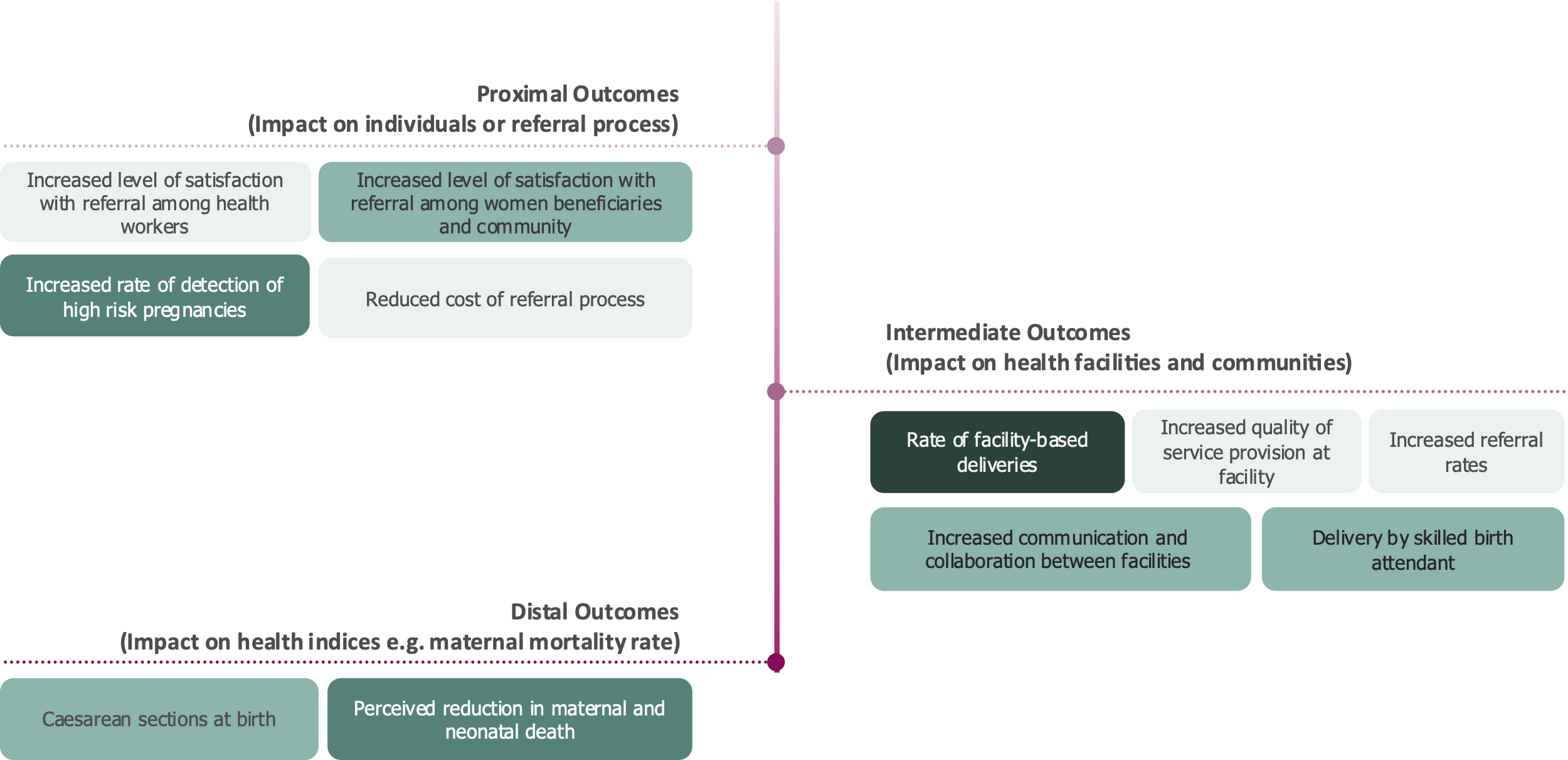


| **Section and Topic** | **Item #** | **Checklist item** | **Location where item is reported** |
| --- | --- | --- | --- |
| **TITLE** | | |  |
| Title | 1 | Identify the report as a systematic review. | Page 1 |
| **ABSTRACT** | | |  |
| Abstract | 2 | See the PRISMA 2020 for Abstracts checklist. | Page 2 |
| **INTRODUCTION** | | |  |
| Rationale | 3 | Describe the rationale for the review in the context of existing knowledge. | Page 3 |
| Objectives | 4 | Provide an explicit statement of the objective(s) or question(s) the review addresses. | Page 3 |
| **METHODS** | | |  |
| Eligibility criteria | 5 | Specify the inclusion and exclusion criteria for the review and how studies were grouped for the syntheses. | Page 4 |
| Information sources | 6 | Specify all databases, registers, websites, organisations, reference lists and other sources searched or consulted to identify studies. Specify the date when each source was last searched or consulted. | Page 3 |
| Search strategy | 7 | Present the full search strategies for all databases, registers and websites, including any filters and limits used. | Supplementary Table S1 |
| Selection process | 8 | Specify the methods used to decide whether a study met the inclusion criteria of the review, including how many reviewers screened each record and each report retrieved, whether they worked independently, and if applicable, details of automation tools used in the process. | Page 4 |
| Data collection process | 9 | Specify the methods used to collect data from reports, including how many reviewers collected data from each report, whether they worked independently, any processes for obtaining or confirming data from study investigators, and if applicable, details of automation tools used in the process. | Page 4 |
| Data items | 10a | List and define all outcomes for which data were sought. Specify whether all results that were compatible with each outcome domain in each study were sought (e.g., for all measures, time points, analyses), and if not, the methods used to decide which results to collect. | Page 4 |
|  | 10b | List and define all other variables for which data were sought (e.g., participant and intervention characteristics, funding sources). Describe any assumptions made about any missing or unclear information. | Page 4 |
| Study risk of bias assessment | 11 | Specify the methods used to assess risk of bias in the included studies, including details of the tool(s) used, how many reviewers assessed each study and whether they worked independently, and if applicable, details of automation tools used in the process. | Page 4 |
| Effect measures | 12 | Specify for each outcome the effect measure(s) (e.g., risk ratio, mean difference) used in the synthesis or presentation of results. | N/A |
| Synthesis methods | 13a | Describe the processes used to decide which studies were eligible for each synthesis (e.g., tabulating the study intervention characteristics and comparing against the planned groups for each synthesis (item #5)). | Page 4 |
|  | 13b | Describe any methods required to prepare the data for presentation or synthesis, such as handling of missing summary statistics, or data conversions. | N/A |
|  | 13c | Describe any methods used to tabulate or visually display results of individual studies and syntheses. | Page 4 |
|  | 13d | Describe any methods used to synthesize results and provide a rationale for the choice(s). If meta-analysis was performed, describe the model(s), method(s) to identify the presence and extent of statistical heterogeneity, and software package(s) used. | Page 4 |
|  | 13e | Describe any methods used to explore possible causes of heterogeneity among study results (e.g. subgroup analysis, meta-regression). | N/A |
|  | 13f | Describe any sensitivity analyses conducted to assess robustness of the synthesized results. | N/A |
| Reporting bias assessment | 14 | Describe any methods used to assess risk of bias due to missing results in a synthesis (arising from reporting biases). | N/A |
| Certainty assessment | 15 | Describe any methods used to assess certainty (or confidence) in the body of evidence for an outcome. | N/A |
| **RESULTS** | | |  |
| Study selection | 16a | Describe the results of the search and selection process, from the number of records identified in the search to the number of studies included in the review, ideally using a flow diagram. | Page 4 (Figure 2) |
|  | 16b | Cite studies that might appear to meet the inclusion criteria, but which were excluded, and explain why they were excluded. | Supplementary Table S4a-d |
| Study characteristics | 17 | Cite each included study and present its characteristics. | Page 4 (Tables 2 and 3) |
| Risk of bias in studies | 18 | Present assessments of risk of bias for each included study. | Page 5 (Table 4) |
| Results of individual studies | 19 | For all outcomes, present, for each study: (a) summary statistics for each group (where appropriate) and (b) an effect estimate and its precision (e.g. confidence/credible interval), ideally using structured tables or plots. | Page 5 (Tables 2 and 3) |
| Results of syntheses | 20a | For each synthesis, briefly summarise the characteristics and risk of bias among contributing studies. | N/A |
|  | 20b | Present results of all statistical syntheses conducted. If meta-analysis was done, present for each the summary estimate and its precision (e.g. confidence/credible interval) and measures of statistical heterogeneity. If comparing groups, describe the direction of the effect. | N/A |
|  | 20c | Present results of all investigations of possible causes of heterogeneity among study results. | N/A |
|  | 20d | Present results of all sensitivity analyses conducted to assess the robustness of the synthesized results. | N/A |
| Reporting biases | 21 | Present assessments of risk of bias due to missing results (arising from reporting biases) for each synthesis assessed. | N/A |
| Certainty of evidence | 22 | Present assessments of certainty (or confidence) in the body of evidence for each outcome assessed. | N/A |
| **DISCUSSION** | | |  |
| Discussion | 23a | Provide a general interpretation of the results in the context of other evidence. | Page 7 |
|  | 23b | Discuss any limitations of the evidence included in the review. | Page 9 |
|  | 23c | Discuss any limitations of the review processes used. | Page 9 |
|  | 23d | Discuss implications of the results for practice, policy, and future research. | Page 9 |
| **OTHER INFORMATION** | | |  |
| Registration and protocol | 24a | Provide registration information for the review, including register name and registration number, or state that the review was not registered. | Page 3 |
|  | 24b | Indicate where the review protocol can be accessed, or state that a protocol was not prepared. | Page 3 |
|  | 24c | Describe and explain any amendments to information provided at registration or in the protocol. | N/A |
| Support | 25 | Describe sources of financial or non-financial support for the review, and the role of the funders or sponsors in the review. | Page 1 |
| Competing interests | 26 | Declare any competing interests of review authors. | Page 1 |
| Availability of data, code and other materials | 27 | Report which of the following are publicly available and where they can be found: template data collection forms; data extracted from included studies; data used for all analyses; analytic code; any other materials used in the review. | N/A |

*From:*  Page MJ, McKenzie JE, Bossuyt PM, Boutron I, Hoffmann TC, Mulrow CD, et al. The PRISMA 2020 statement: an updated guideline for reporting systematic reviews. BMJ 2021;372:n71. doi: 10.1136/bmj.n71

For more information, visit: <http://www.prisma-statement.org/>
